# A Caged Neutral 17-Valence-Electron Iron(I) Radical [Fe(CO)_2_(Cl)(P((CH_2_)_10_)_3_P)]^•^: Synthetic, Structural, Spectroscopic, Redox, and Computational Studies

**DOI:** 10.1021/acs.inorgchem.4c02275

**Published:** 2024-08-20

**Authors:** Samuel R. Zarcone, Zihan Zhang, Suhashini Handunneththige, Zhen Ni, Nattamai Bhuvanesh, Michael Nippe, Karsten Meyer, Michael B. Hall, John A. Gladysz

**Affiliations:** † Department of Chemistry, Texas A&M University, P.O. Box 30012, College Station, Texas 77842-3012, United States; ‡ Department of Chemistry and Pharmacy, Inorganic Chemistry, Friedrich-Alexander-Universität Erlangen-Nürnberg (FAU), Egerlandstraße 1, 91058 Erlangen, Germany

## Abstract

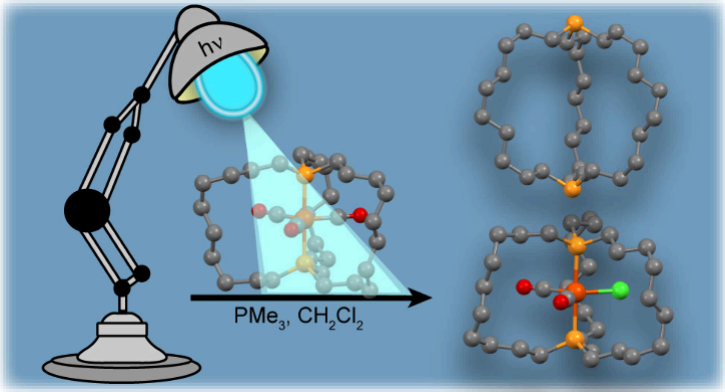

UV irradiation of yellow CH_2_Cl_2_ solutions of *trans*-Fe(CO)_3_(P((CH_2_)_10_)_3_P) (**2a**) and PMe_3_ (10 equiv) gives, in addition to the previously reported dibridgehead diphosphine P((CH_2_)_10_)_3_P (46%), a green paramagnetic complex that crystallography shows to be the trigonal-bipyramidal iron(I) radical *trans*-[Fe(CO)_2_(Cl)(P((CH_2_)_10_)_3_P)]^•^ (**1a**^•^; 31% after workup). This is a rare example of an isolable species of the formula [Fe(CO)_4–*n*_(L)_*n*_(X)]^•^ (*n* = 0–3, L = two-electron-donor ligand; X = one-electron-donor ligand). Analogous precursors with longer P(CH_2_)_*n*_P segments (*n* = 12, 14, 16, 18) give only the demetalated diphosphines, and a rationale is proposed. The magnetic susceptibility of **1a**^•^, assayed by Evans’ method and SQUID measurements, indicates a spin (*S*) of ^1^/_2_. Cyclic voltammetry shows that **1a**^•^ undergoes a partially reversible one-electron oxidation, but no facile reduction. The UV–visible, EPR, and ^57^Fe Mössbauer spectra are analyzed in detail. Complex **2a** is similarly studied, and, despite the extra valence electron, exhibits a comparable oxidation potential (Δ*E*_1/2_ ≤ 0.04 V). The crystal structure shows a cage conformation, solvation level, disorder motif, and unit cell parameters essentially identical to those of **1a**^•^. DFT calculations provide much insight regarding the structural, redox, and spectroscopic properties.

## Abstract 2

Irradiation of a CH_2_Cl_2_ solution of the Fe(CO)_3_ complex that is encased in a triply *trans*-spanning diphosphine cage affords the title radical, which is a rare example of a stable pentacoordinate iron(I) species. The structures of the radical and its precursor are compared crystallographically and computationally. The oxidation potentials are surprisingly close, although molecular orbital analyses provide insight. The radical is further characterized by UV−visible, SQUID, EPR, and Mössbauer measurements.

## Introduction

**Figure 1:**
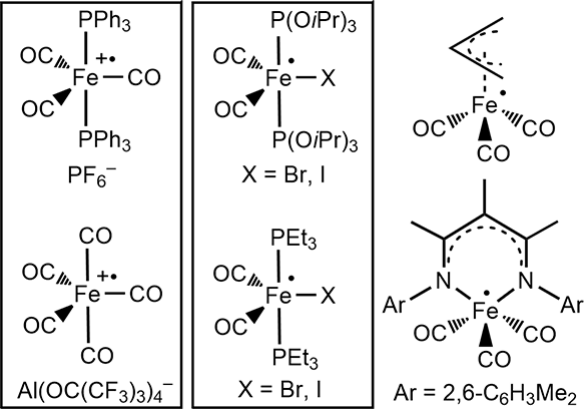
Some previously isolated pentacoordinate iron(I) carbonyl radicals.

For a variety of reasons, the synthesis, isolation, and study of organometallic radicals has lagged behind that of diamagnetic species.^1^ This dichotomy also extends to computational investigations.^2,3^ Among the many classes of interest, considerable attention has been given to 17-valence-electron pentacoordinate iron(I) carbonyl radicals (Figure 1). Teams spearheaded by Connelley, Baird, and Krossing have reported the synthesis and extensive spectroscopic and structural characterization of the cationic complexes *trans*-[Fe(CO)_3_(PPh_3_)_2_]^•+^PF_6_^–^ and [Fe(CO)_5_]^•+^Al(OC(CF_3_)_3_)_4_^–^.^4,5^ Berke has isolated neutral adducts of the formula *trans*-[Fe(CO)_2_(X)(PX′_3_)_2_]^•^ (X/X′ = O*i*Pr/Br, O*i*Pr/I, Et/Br, Et/I),^6^ and related species have been detected in situ.^6−8^ Additional isolable neutral pentacoordinate iron carbonyl radicals are illustrated in Figure 1.^9^

Given the innate reactivity of most types of radicals, chemists often seek to sterically shield them. In this context, the P(O*i*Pr)_3_-substituted radicals in Figure 1 are much more stable than the P(OMe)_3_ homologues,^6^ in accord with the greater phosphite ligand cone angle. Similar relationships have been established for numerous pairs of organometallic radicals.^6,10^ Some recent dramatic examples involve bulky *meta*-terphenyl isocyanide ligands that contain multiple isopropyl or trifluoromethyl substituents, which enable the crystallization of the pentacoordinate Group 7 complexes [M(CO)(CNAr)_4_]^•^ (M = Mn, Tc, Re).^10^ However, comparably enveloping steric environments have not yet been applied to pentacoordinate Group 8 radicals.

In this paper, we report the (1) serendipitous isolation, (2) structural, spectroscopic, and electrochemical characterization, and (3) computational analysis of the neutral pentacoordinate iron dicarbonyl chloride radical *trans*-[Fe(CO)_2_(Cl)(P((CH_2_)_10_)_3_P)]^•^ (**1a**^•^). The stability is attributed to a new motif of steric protection afforded by a cage-like, triply *trans*-spanning diphosphine ligand. Furthermore, the mechanistic sequences involved are believed to involve topologically unusual steps tantamount to turning a molecule inside out. The many unique physical properties of **1a**^•^ are thoroughly interpreted, often with the aid of the DFT calculations.

## Results

### Synthesis of the Title Complex

**Scheme 1:**
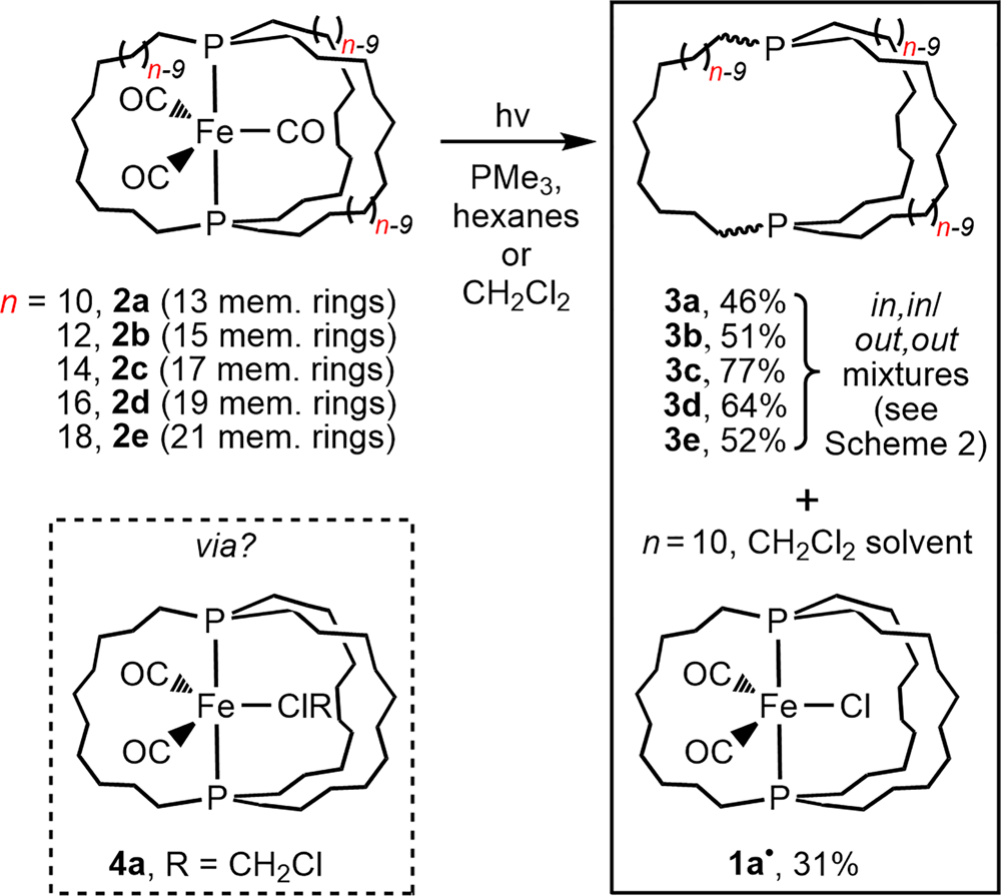
Photochemical Conversions of **2a**–**e** to Dibridgehead Diphosphines **3a**–**e** and/or the Iron(I) Radical **1a**^•^

**Scheme 2:**
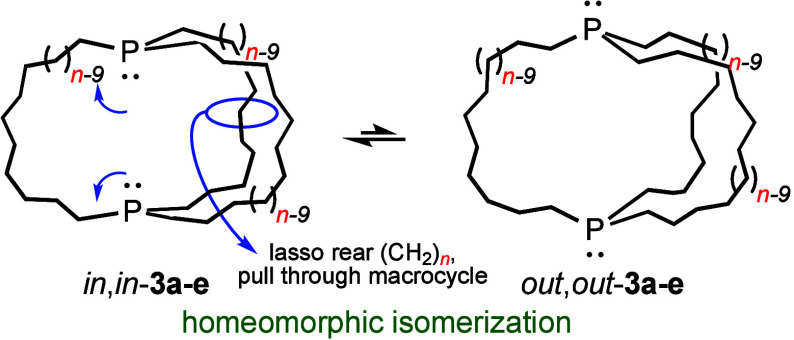
Homeomorphic Isomerization of Dibridgehead Diphosphines **3a**–**e**

The iron tricarbonyl complexes **2** in Scheme 1 have been under study in the group of one author for some time.^11^ They feature *trans*-spanning diphosphines with three P(CH_2_)_*n*_P tethers, the lengths of which can be varied. It was recently found that irradiation with a Hanovia mercury lamp (450 W) in the presence of excess PMe_3_ in hexanes or CH_2_Cl_2_ afforded the corresponding free dibridgehead diphosphines **3**.^12,13^ These could be isolated as white solids in 46–77% yields. All are capable of homeomorphic isomerization, a topological process that turns the molecules inside out, equilibrating *in,in* and *out,out* isomers without the need for pyramidal inversion of the phosphorus bridgeheads. This is illustrated in Scheme 2, and a video is available in the Supporting Information of a recent publication.^14^

As further elaborated on in the Discussion section, the Fe(CO)_3_ moiety is believed to escape by an initial iron–phosphorus bond cleavage, followed by homeomorphic isomerization of the diphosphine to give an adduct of *out,out*-**3** and Fe(CO)_3_. Subsequent attack of PMe_3_ would displace the η^1^-diphosphine from iron. Although a small amount of *trans*-Fe(CO)_3_(PMe_3_)_2_ can be detected during photolyses, the conversion never exceeds 5%, presumably because this complex is itself photoactive under these conditions.

When **2b**–**e** were photolyzed in hexane or CH_2_Cl_2_, the yellow solutions became cloudy orange.^15^ However, CH_2_Cl_2_ solutions of **2a** turned green.^16^ Workup afforded a paramagnetic green material in 31% yield based upon the structure established below (**3a** was also produced). The IR spectrum exhibited two strong ν_CO_ bands (1944 and 1861 cm^–1^) at higher frequencies than those of the precursor (1853 and 1841 cm^–1^), and microanalysis supported the formulation *trans*-[Fe(CO)_2_(Cl)(P((CH_2_)_10_)_3_P)]^•^ (**1a**^•^). A mass spectrum showed a strong ion corresponding to [M – 2(CO)]^+^. As illustrated in Figure s12, solid **1a**^•^ decomposed over the course of 20 h in air. Solutions decomposed over the course of 1.5 h.

### Crystallography

**Figure 2:**
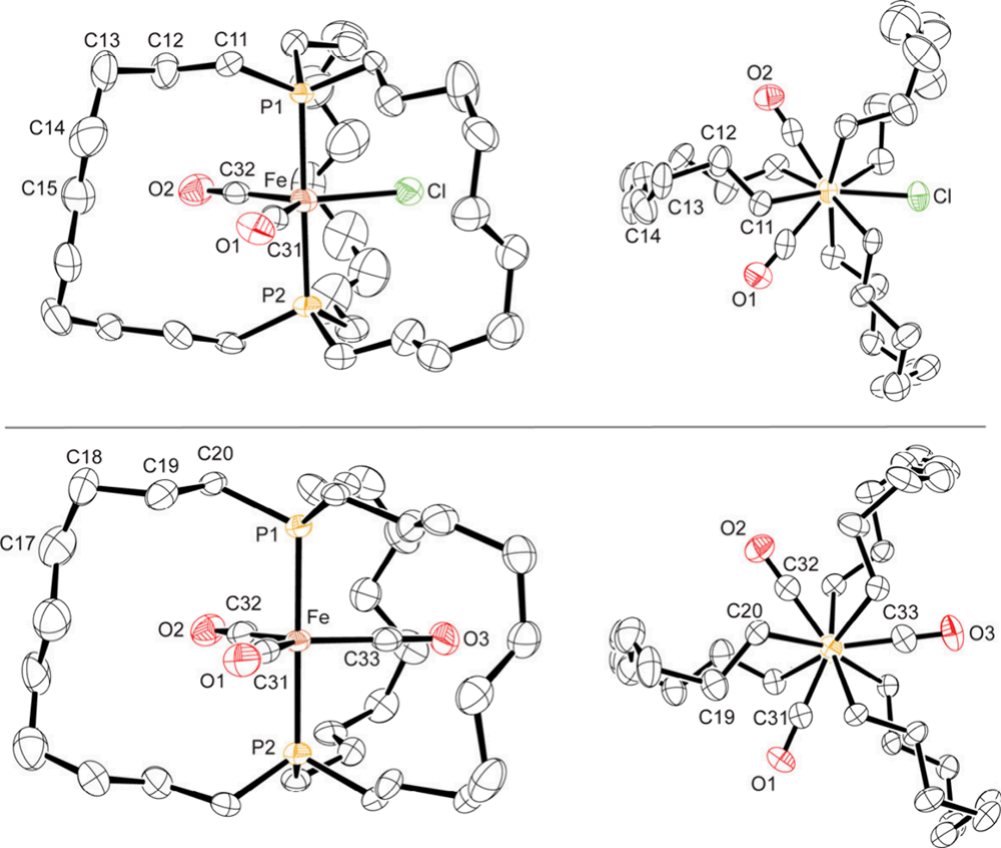
Thermal ellipsoid plots of the molecular structures of **1a**^•^·0.5C_6_H_14_ (top) and **2a**·0.45C_6_H_14_ (bottom) (50% probability levels, dominant conformations, hydrogen and solvate atoms omitted).

Single crystals of a hexane hemisolvate of **1a**^•^ could be grown, and the X-ray structure was solved as described in Table 1 and the Experimental Section. The (CH_2_)_10_ segments exhibited two conformations, which could be modeled by a 59:41 occupancy ratio. Thermal ellipsoid plots of the dominant isomer are provided in Figure 2, and bond lengths and angles about iron are summarized in Table 2. The trigonal-bipyramidal geometry was evidenced by a P–Fe–P angle of 178.53(5)°, P–Fe–CO and P–Fe–Cl angles ranging from 89.65(7)° to 91.64(7)°, and OC–Fe–CO and Cl–Fe–CO angles ranging from 107.5(1)° to 126.57(8)°.

**Table 1:** Summary of Crystallographic Data

	**1a**^•^·0.50C_6_H_14_	**2a**·0.45C_6_H_14_
empirical formula	C_35_H_67_ClFeO_2_P_2_	C_35.7_H_66.3_FeO_3_P_2_
formula weight	673.12	661.37
temperature [K]	110.0	100.00
diffractometer	Bruker Venture	XtaLAB Synergy, Dualflex, HyPix
wavelength [Å]	0.71073	1.54184
crystal system	monoclinic	monoclinic
space group	*P*2_1_/*c*	*P*2_1_/*c*
unit cell dimensions		
*a* [Å]	11.4017(6)	11.45057(5)
*b* [Å]	11.7236(6)	11.76785(6)
*c* [Å]	27.9438(14)	28.05972(13)
α [deg]	90	90
β [deg]	96.4986(19)	96.6447(4)
γ [deg]	90	90
*V* [Å^3^]	3711.2(3)	3755.61(3)
*Z*	4	4
ρ_calc_ [Mg m^–3^]	1.205	1.170
μ [mm^–1^]	0.593	4.252
*F*(000)	1464	1422
crystal size [mm^3^]	0.249 × 0.082 × 0.048	0.44 × 0.28 × 0.12
θ limit [deg]	1.467–25.729	3.171–70.073
index range (*h*, *k*, *l*)	–13/13, –14/13, –34/34	–14/14, –13/13, –35/35
reflections collected	70838	69531
independent reflections	7060	7106
*R*(int)	0.0493	0.0350
completeness to θ [%]	100.0	99.8
max and min transmission	0.4684 and 0.2960	1.000 and 0.225
data/restraints/parameters	7060/1284/534	7106/159/633
goodness-of-fit on *F*^2^	0.866	1.052
*R* indices (final) [*I* > 2σ(*I*)]		
*R*_1_	0.0779	0.0377
*wR*_2_	0.1813	0.0938
*R* indices (all data)		
*R*_1_	0.0794	0.0389
*wR*_2_	0.1824	0.0947
largest diff peak and hole [e Å^–3^]	0.864 and −0.534	0.579 and −0.287

**Table 2:** Selected Crystallographic Distances (Å) and Angles (deg) for **1a**^•^ and **2a** and the Corresponding Computational Data for **1a′**^•^, **2a′**, and PMe_3_-Substituted **7′**^•^, **7′**^–^, and **8′**

	**1a**^•^·0.5C_6_H_14_	**1a′**^•^	**2a**·0.45C_6_H_14_	**2a′**	**7′**^•^	**7′**^–^	**8′**
P···P	4.517(1)	4.61	4.4323(7)	4.52	4.52	4.39	4.45
Fe–P(1)	2.2603(10)	2.31	2.2199(5)	2.26	2.27	2.20	2.23
Fe–P(2)	2.2577(11)	2.31	2.2128(5)	2.27	2.27	2.20	2.23
Fe–C(31)	1.793(5)	1.78	1.764(2)	1.77	1.79	1.73	1.77
Fe–C(32)	1.802(5)	1.79	1.763(2)	1.77	1.79	1.73	1.77
Fe–C(33)			1.768(2)	1.77			1.77
Fe–Cl	2.3128(12)	2.36			2.36	2.60	
Fe–O(1)	2.866(4)	2.94	2.922(1)	2.93	2.94	2.89	2.93
Fe–O(2)	2.897(4)	2.94	2.925(2)	2.93	2.94	2.89	2.93
Fe–O(3)			2.928(2)	2.93			2.93
C(31)–O(1)	1.074(5)	1.15	1.157(2)	1.16	1.15	1.18	1.16
C(32)–O(2)	1.095(5)	1.16	1.162(2)	1.16	1.15	1.18	1.16
C(33)–O(3)			1.160(2)	1.16			1.16
C(1)–P(1)^a^	1.814(5)	1.86	1.903(4)	1.86	1.84	1.85	1.84
C(2)–P(1)^a^	1.843(5)	1.86	1.837(4)	1.86	1.84	1.85	1.84
C(3)–P(1)^a^	1.850(6)	1.85	1.804(4)	1.86	1.84	1.85	1.84
C(4)–P(2)^a^	1.835(6)	1.86	1.878(5)	1.86	1.84	1.85	1.84
C(5)–P(2)^a^	1.839(6)	1.86	1.821(5)	1.86	1.84	1.85	1.84
C(6)–P(2)^a^	1.821(7)	1.85	1.768(4)	1.86	1.84	1.85	1.84
P(1)–Fe–P(2)	178.53(5)	176.3	178.18(2)	176.9	173.0	170.8	179.9
P(1)–Fe–C(31)	89.7(1)		89.52(6)				
P(1)–Fe–C(32)	91.0(1)		91.31(6)				
P(1)–Fe–C(33)			89.98(6)				
P(1)–Fe–Cl	89.89(4)	88.76			86.48	85.38	
P(2)–Fe–Cl	89.83(4)	88.47			86.48	85.38	
P(2)–Fe–C(31)	89.3(2)		88.94(6)				
P(2)–Fe–C(32)	90.3(1)		90.31(6)				
P(2)–Fe–C(33)			89.97(6)				
C(32)–Fe–C(31)	108.3(2)		120.63(9)				
C(32)–Fe–C(33)			118.55(10)				
C(33)–Fe–C(31)			120.82(9)				
C(31)–Fe–Cl	126.2(2)						
C(32)–Fe–Cl	125.5(1)						

a For these distances, the carbon atom numbers differ from those in the CIF file.

Complex **2a**, which differs from **1a**^•^ only by a CO/Cl replacement (identical tether lengths), was viewed as an especially valuable reference compound. Accordingly, the crystal structure of a hexane solvate of **2a** was solved. Again, the (CH_2_)_10_ segments exhibited two conformations, which in this case was best modeled by a 54:46 occupancy ratio. The thermal ellipsoid plots are presented side-by-side with those of **1a**^•^ in Figure 2. As can be seen in Table 1, the space group, crystal system, and *Z* value of **2a** and **1a**^•^ are identical (*P*2_1_/*c*, monoclinic, 4), and the unit cell dimensions vary by less than 0.4%, showing the lattices to be essentially isostructural.^17^

As would be intuitively expected from the reduced number of valence electrons and presumably attenuated back-bonding in **1a**^•^, the Fe–C bonds [1.793(5) and 1.802(5) Å] are longer than those of **2a** [1.764(2), 1.763(2), and 1.768(2) Å], **2c** [1.761(3) and 2 × 1.764(2) Å], and other closely related 18-valence-electron Fe(CO)_3_ adducts.^11a^ The CO bond lengths [1.074(5) and 1.095(5) Å] are in turn shorter [**2a**, 1.157(2), 1.160(2), and 1.162(2) Å; **2c**, 1.162(3) and 2 × 1.164(3) Å]. It merits note in passing that complexes of the formula Fe(CO)_3_(PPh_2_R)_2_ can additionally adopt square pyramidal geometries,^18^ and that isomerization can take place upon oxidation.^19^

### Additional Physical Characterization

**Figure 3:**
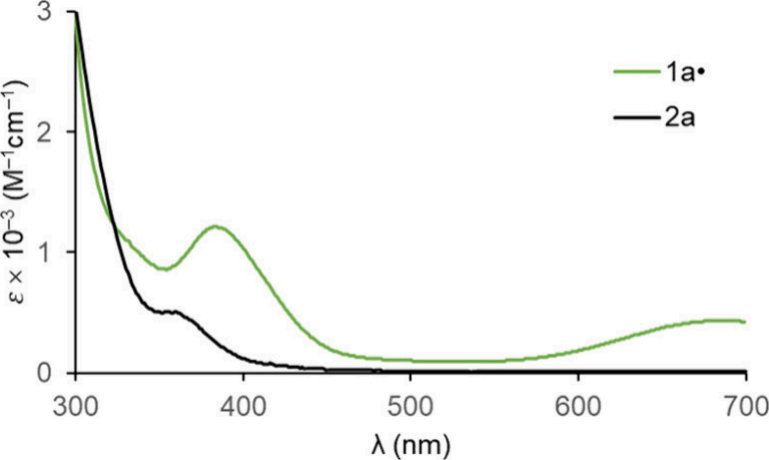
UV–visible spectra (8.50–8.60 × 10^–5^ M in CH_2_Cl_2_) of **1a**^•^ (λ_max_ (nm) [ε (M^–1^ cm^–1^)]: 382 [1,230], 693 [480] and **2a** (λ_max_ (nm) [ε (M^–1^ cm^–1^)]: 359 [510]).

UV–visible spectra of **1a**^•^ and **2a** were recorded in CH_2_Cl_2_. As depicted in Figure 3, **1a**^•^ exhibits a moderately intense band at 382 nm (ε 1230 M^–1^ cm^–1^) superimposed on an absorption tail, and a broader and weaker band at 692 nm (ε 490 M^–1^ cm^–1^). The latter is of course primarily responsible for the green color. The precursor **2a** displays a shoulder of modest intensity at 359 nm (ε 510 M^–1^ cm^–1^), and did not absorb above 450 nm. The underlying electronic transitions are defined with the aid of TD-DFT calculations below.

**Figure 4:**
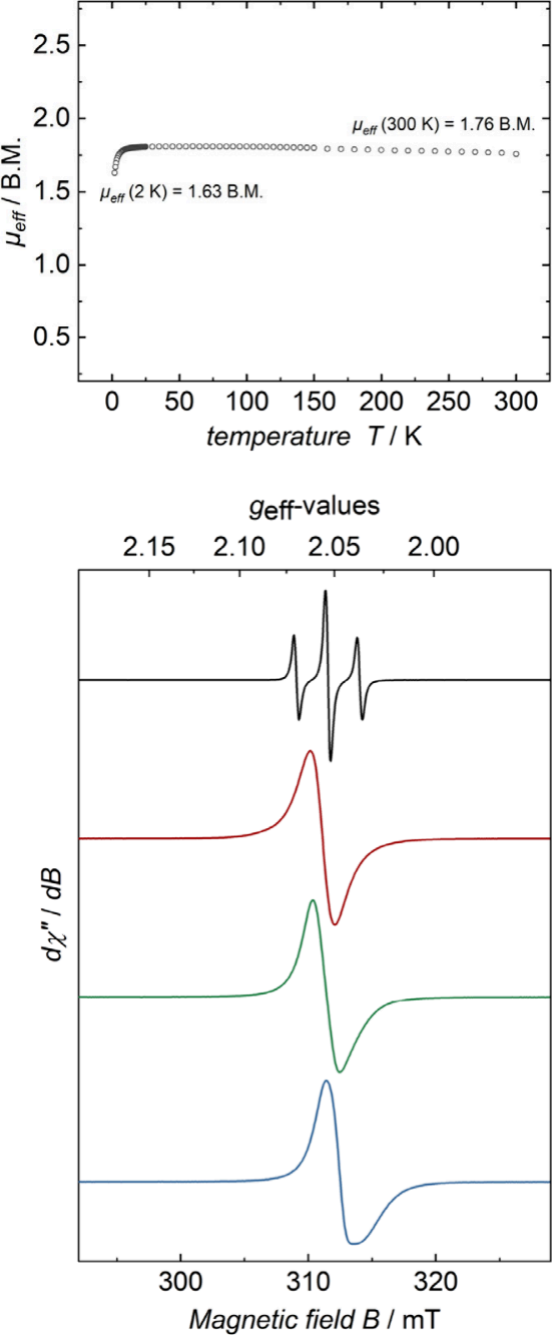
Top: VT SQUID magnetization data for **1a**^•^; Bottom: CW X-band EPR spectrum of a 3 mM CH_2_Cl_2_ solution of **1a**^•^ at 293 K (black trace), an analogous frozen solution at 95 K (red trace), a powdered sample at 95 K (green trace), and a crystalline sample at 95 K (blue trace). The simulation of the liquid solution data revealed the following EPR parameters: g_iso_ = 2.05 and A_iso_ = (^31^P, *n* = 2, *I* = ^1^/_2_, 100%) 23.7 × 10^–4^ cm^–1^ (Figure s2).

Next, the magnetic susceptibility (χ) of **1a**^•^ was determined by Evans’ method. The value, μ_eff_ 1.67 μ_B_, is typical of complexes with a spin (*S*) of ^1^/_2_. Two VT SQUID measurements were made with powdered samples. Both gave identical results, as depicted in Figure 4 (top), indicating a magnetic moment (μ_B_) ranging from 1.76 (300 K) to 1.63 (2 K).

As shown in Figure 4 (bottom), CW X-band EPR spectra of **1a**^•^ were recorded under several conditions. An isotropic spectrum in liquid CH_2_Cl_2_ at 293 K (black trace) exhibited a triplet due to ^31^P coupling with a g_iso_ value of 2.05 and a superhyperfine coupling constant (A_iso_) of 2.48 mT (23.7 × 10^–4^ cm^–1^). A spectrum of the corresponding frozen glass (95 K) also gave a signal with a g_iso_ value of 2.05, but without coupling (red trace).

**Figure 5:**
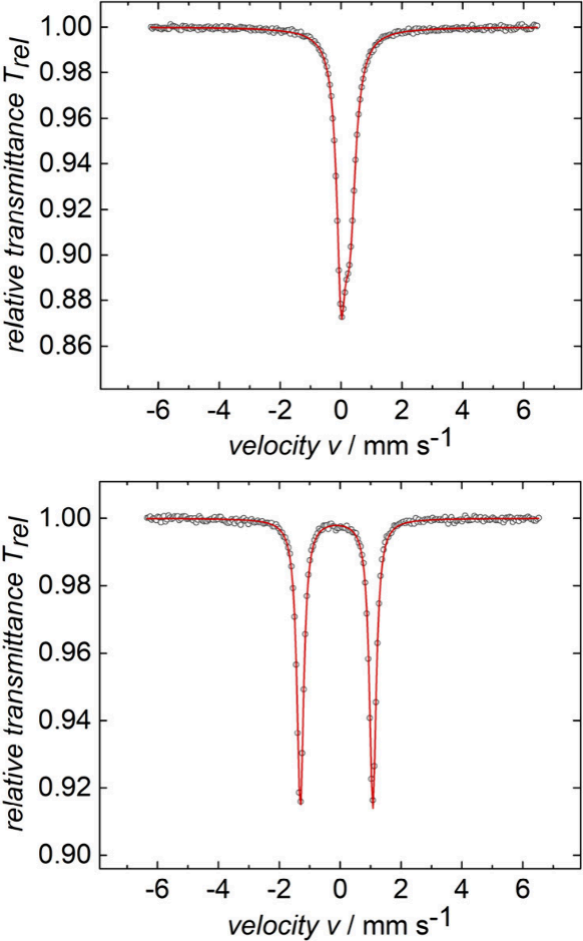
Zero field ^57^Fe Mössbauer spectrum of solid **1a**^•^ (top) and **2a** (bottom) at 77 K and referenced to α-iron. Collected data are represented by black circles, and additional data are in Tables 3 and 4.

Next, zero-field ^57^Fe Mössbauer spectra of solid samples of **1a**^•^ and **2a** were recorded at 77 K. As shown in Figure 5 (top), **1a**^•^ exhibits a positive isomer shift (IS or δ) of 0.14 mm s^–1^, a conspicuously small quadrupole splitting (QS) of 0.29 mm s^–1^, and a line width (Γ) of 0.34 mm s^–1^. In contrast, **2a** (bottom) displays a negative isomer shift of −0.12 mm s^–1^, a larger quadrupole splitting of 2.38 mm s^–1^, and a line width of 0.27 mm s^–1^. These properties are summarized in Tables 3 and 4, together with the experimental data from the literature^20−22^ and DFT computational results^23^ described and interpreted below.

**Table 3:** Computed and Experimental ^57^Fe Mössbauer Quadrupole Splittings (QS) of Selected Complexes

	complex	calc. QS (mm s^–1^)	exp. QS (mm s^–1^)
**1a**^•^	*trans*-[Fe(CO)_2_(Cl)(P((CH_2_)_10_)_3_P)]^•^	0.74	0.29
**2a**	*trans*-Fe(CO)_3_(P((CH_2_)_10_)_3_P)	2.55	2.38
**7′**^•^	*trans*-[Fe(CO)_2_(Cl)(P(CH_3_)_3_)_2_]^•^	0.71	
**7′**^–^	*trans*- [Fe(CO)_2_(Cl)(P(CH_3_)_3_)_2_]^−^	2.33	
**8′**	*trans*-Fe(CO)_3_(P(CH_3_)_3_)_2_	2.60	
	*trans*-Fe(CO)_3_(PCy_3_)_2_		2.75^21^
	*trans*-Fe(CO)_3_(PPh_3_)_2_	3.01	2.76^20^
	Fe(CO)_5_	2.30	2.55^5^
	[Fe(CO)_5_]^•+^Al(OC(CF_3_)_3_)_4_^–^	0.47^a^	0.53^5^

a Calculated for the cation Fe(CO)_5_^•+^.

**Table 4:** Computed and Experimental ^57^Fe Mössbauer Isomer Shifts (IS) of Selected Complexes

	complex	electron density (a.u.^–3^)	calc. IS (mm s^–1^)	exp. IS (mm s^–1^)
**1a**^•^	*trans*-[Fe(CO)_2_(Cl)(P((CH_2_)_10_)_3_P)]^•^	11606.45	0.12	0.14
**2a**	*trans*-Fe(CO)_3_(P((CH_2_)_10_)_3_P)	11607.58	–0.14	–0.12
**7′**^•^	*trans*-[Fe(CO)_2_(Cl)(P(CH_3_)_3_)_2_]^•^	11606.31	0.16	
**7′**^–^	*trans*-[Fe(CO)_2_(Cl)(P(CH_3_)_3_)_2_]^−^	11606.87	0.02	
**8′**	*trans*-Fe(CO)_3_(P(CH_3_)_3_)_2_	11607.48	–0.12	
	*trans*-Fe(CO)_3_(PCy_3_)_2_			–0.125^21,22^
TR1^a^	*trans*-Fe(CO)_3_(PPh_3_)_2_	11607.47	–0.12	–0.105^20,22^
				–0.151^21,22^
TR2^a^	Fe(CO)_5_	11607.36	–0.09	–0.08^5^
TR3^a^	[Fe(CO)_5_]^•+^Al(OC(CF_3_)_3_)_4_^–^	11606.25^b^	0.17	0.17^5^

a TR1,TR2, and TR3 are training sets. Parameters for the isomer shift (δ) were calculated using the equation δ = α[ρ(0) – *C*] + β, where α, β, and *C* are constants and ρ(0) is the electron density.^23^ The electron densities of the compounds comprising the training set were graphed versus their experimental isomer shifts, resulting in α = −0.2356 and β = 0.2296 (*C* was set at a constant of 11606.00).

b Calculated for the cation Fe(CO)_5_^•+^.

**Figure 6:**
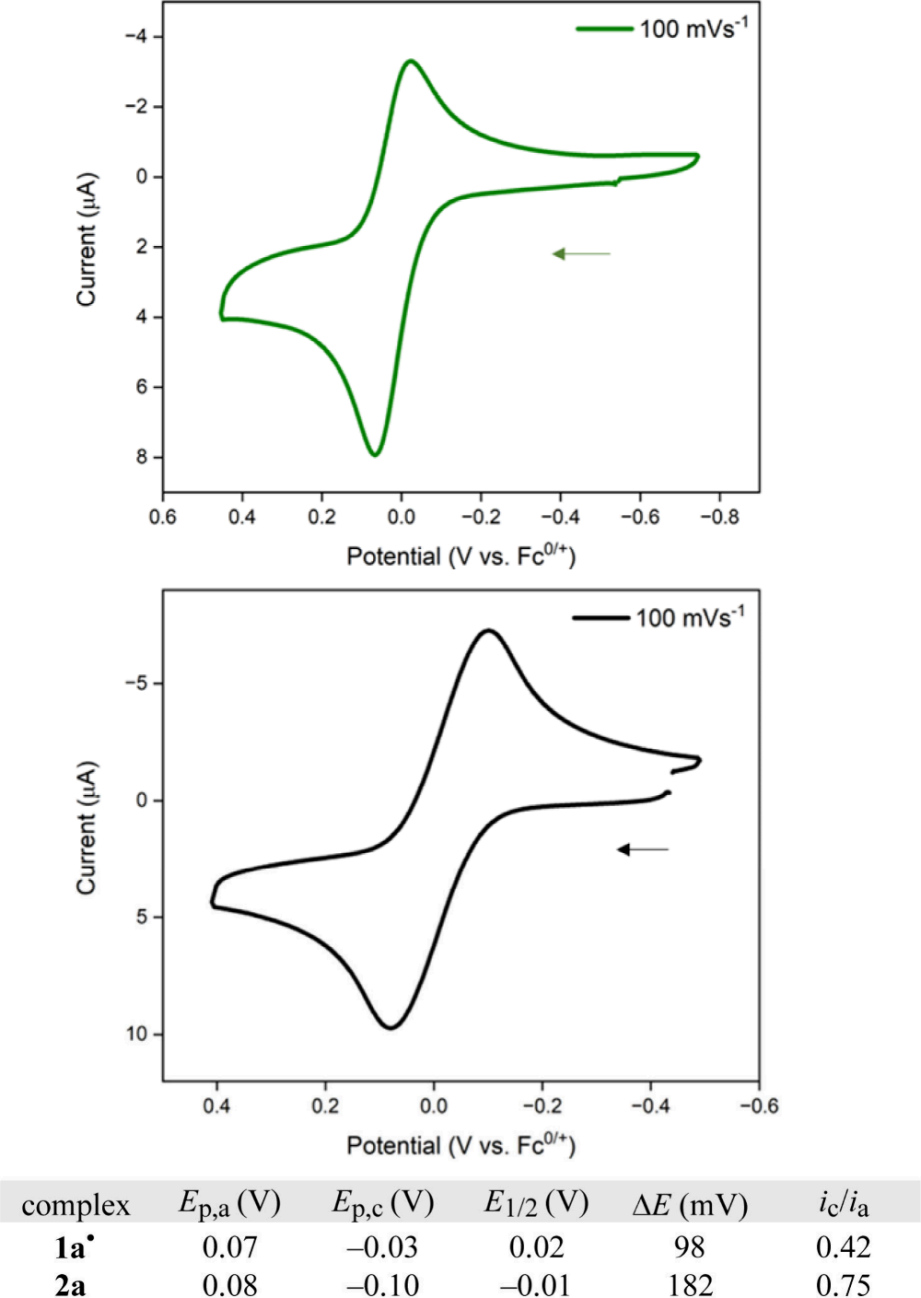
Cyclic voltammograms of **1a**^•^ (top) and **2a** (bottom). Conditions: 1.0 mM in 0.10 M *n*-Bu_4_N^+^PF_6_^–^/CH_2_Cl_2_ under argon, 23 ± 1 °C; 3 mm glassy carbon working electrode, Pt wire counter electrode, and Ag wire reference electrode; scan rate, 100 mV s^–1^. All scans were continued to −2.0 and 1.0 V, but no additional features were observed.

Cyclic voltammograms of **1a**^•^ and **2a** are depicted in Figure 6. These were recorded in CH_2_Cl_2_ under the standard conditions summarized in the caption. Each gave a partially reversible oxidation with *i*_c_/*i*_a_ values of 0.42 and 0.75, respectively. The *E*_1/2_ values (vs Fc^0/+^) show that the oxidation of **1a**^•^, which would give the 16-valence-electron species **1a**^+^, is only slightly less thermodynamically favorable (with respect to any arbitrary oxidizing agent) than that of **2a** to give **2a**^•+^ (0.02 vs −0.01 V; Δ*E*_1/2_ = 0.03 V). A replicate determination on another apparatus but at 200 instead of 100 mV s^–1^ gave *i*_c_/*i*_a_ values of 0.70 and 0.88 and a Δ*E*_1/2_ of 0.04 V (Figure s10). Surprisingly, neither complex underwent reduction when scans were extended to −2.0 V. Computational insight is provided below.

### Computational Characterization

To help interpret the preceding data, DFT calculations were carried out using standard functionals and protocols extensively benchmarked for the electrochemical and Mössbauer calculations, as detailed in the Experimental Section. For computational structures and data, molecules are designated with primes (**1a′**^•^ and **2a′**). To provide additional perspective, three acyclic model complexes with PMe_3_ ligands were also studied: the 17-valence-electron radical *trans*-[Fe(CO)_2_(Cl)(PMe_3_)_2_]^•^ (**7′**^•^), the corresponding 18-valence-electron anion *trans*-[Fe(CO)_2_(Cl)(PMe_3_)_2_]^−^ (**7′**^–^), and the 18-valence-electron adduct *trans*-Fe(CO)_3_(PMe_3_)_2_ (**8′**).^24^ The first two represent experimentally unknown compounds, although **7′**^•^ has been proposed as a transient,^7^ as described in the Discussion section.

**Figure 7:**
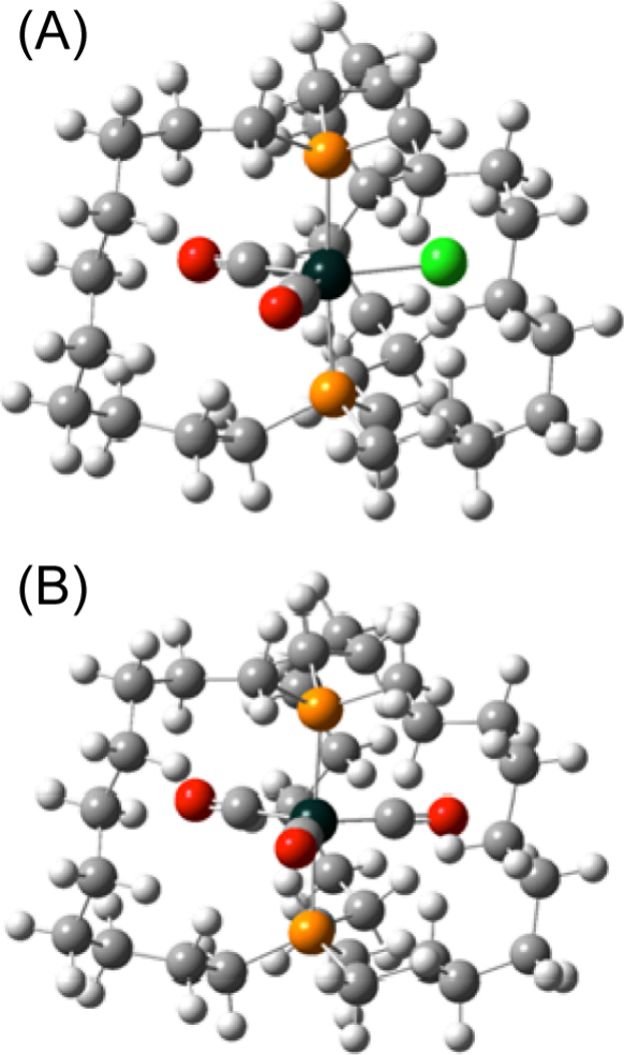
DFT-optimized structures for (A) *trans*-[Fe(CO)_2_(Cl)P((CH_2_)_10_)_3_P]^•^ (**1a′**^•^) and (B) *trans*-Fe(CO)_3_P((CH_2_)_10_)_3_P (**2a′**).

**Figure 8:**
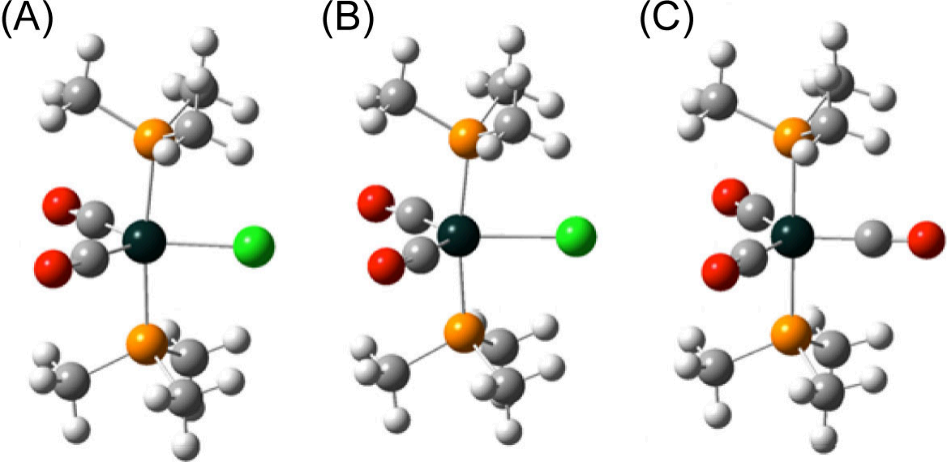
DFT-optimized structures for (A) *trans*-[Fe(CO)_2_(Cl)(P(CH_3_)_3_)_2_]^•^ (**7′**^•^), (B) *trans*-[Fe(CO)_2_(Cl)(P(CH_3_)_3_)_2_]^−^ (**7′**^–^), and (C) *trans*-Fe(CO)_3_(P(CH_3_)_3_)_2_ (**8′**).

As illustrated in Figure 7, gas phase energy minimization afforded structures of **1a′**^•^ and **2a′** that were very close to those obtained crystallographically. Bond lengths and angles associated with the central iron atoms and carbonyl ligands are incorporated into Table 2, and the excellent agreement is apparent. The conformations along the P–Fe–P axes (Figure 2, right) were also nicely reproduced. Figure 8 shows that the corresponding PMe_3_-substituted model compounds **7′**^•^ and **8′** have very similar structures, but now with perfectly staggered P–Fe–P conformations. Interestingly, the anionic 18-electron complex [Fe(CO)_2_(Cl)(P(CH_3_)_3_)_2_]^−^ (**7′**^–^) has a much longer Fe–Cl bond than **7′**^•^ (2.60 vs 2.36 Å), while the other bonds to iron are somewhat shorter (0.06–0.07 Å).

**Figure 9:**
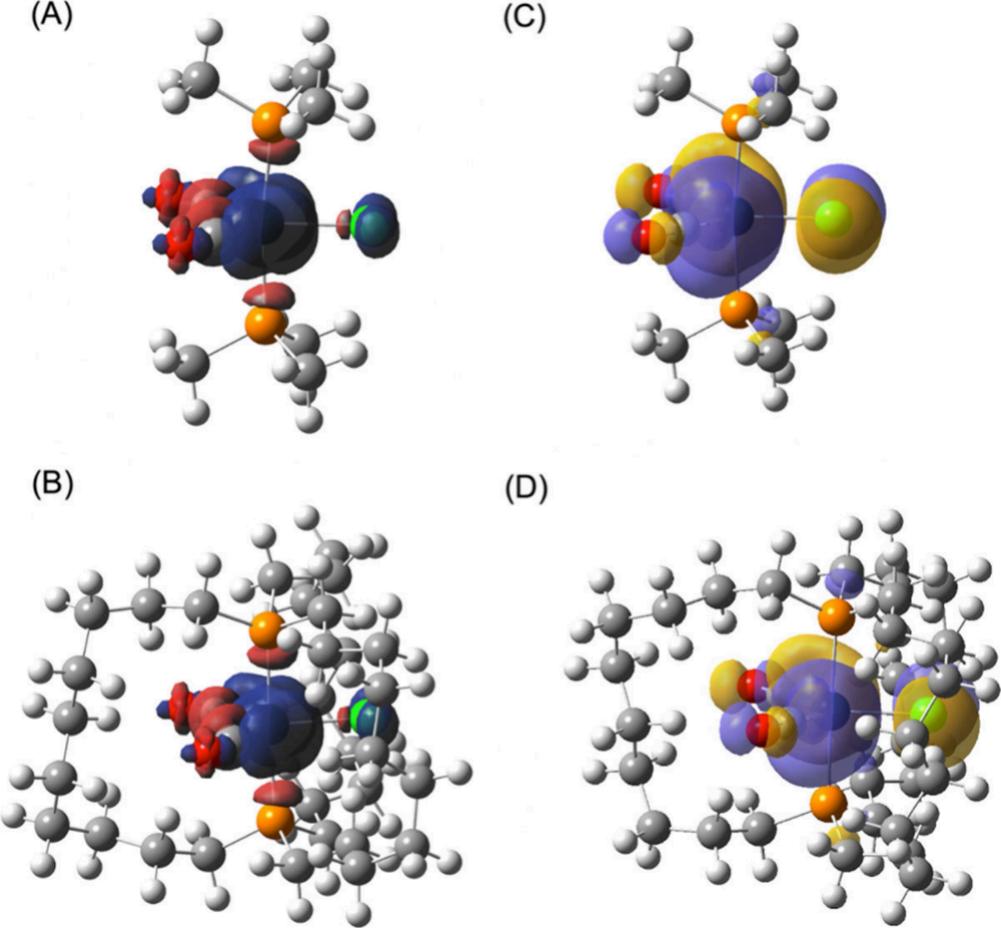
Spin density diagrams for *trans* radical complexes (A) **7′**^•^ and (B) **1a′**^•^ (left). α-HOMO diagrams for (C) **7′**^•^ and (D) **1a′**^•^ (right).

To help understand the Fe–Cl bond length trends, the spin densities of **7′**^•^ and **1a′**^•^ were calculated. As can be derived from the former (Figure 9A), the electron lost from **7′**^–^ comes from the iron 3d_*xy*_ orbital, which is antibonding with respect to the chlorine 3p_*x*_ orbital (*x*-axis perpendicular to the FeP_2_Cl plane). The spin density motif calculated for the full molecule **1a′**^•^ (Figure 9B) is, as expected, very close to that of **7′**^•^. Parts C and D of Figure 9 show the corresponding plot for the HOMO. As expected, the spin density is dominated by the unpaired electron in this orbital, but the spin density shows spin polarization with the opposite spin perpendicular to the majority spin.

A reviewer noted that the experimental Fe–P bonds in **1a**^•^ were longer than those in **2a**, although one might have expected a shortening due to the oxidized iron having a shorter bond radius in **1a**^•^, and asked if this lengthening was due to decreased π-bonding. While parallel trends have been observed with other complexes of iron and phosphorus donor ligands where the corresponding radical cations have been crystallographically characterized^25^ a more thorough examination of this issue was undertaken as this same trend is reproduced by the calculations for both the actual complexes, **1a′**^•^ and **2a′**, and the simplified model complexes **7′**^•^ and **8′**. The Quantum Theory of Atoms in Molecules (QTAIM)^26^ provides a quantum mechanically accurate method to calculate atomic charges. The QTAIM results for **8′** and **7′**^•^ (Table s1) show that, in spite of **7′**^•^ having a higher formal oxidation state than **8′**, the atomic charges differ by less than 0.10 electrons. This small change occurs because the increased donation from Cl, a strong σ and π donor, offsets the expected electron loss from the increase in formal oxidation state. Thus, in spite of the increase in formal oxidation state, the bond radius of the iron should be similar. One can gain some insight into the evolution of the π-bonding by examining the bond lengths. Replacing a CO in **8′** with Cl^–^ to produce **7′**^–^ causes a substantial increase in the back-bonding of the other ligands and both the Fe–C and Fe–P bond lengths decrease substantially and the C–O and P–C bond lengths increase substantially, a characteristic signature of increased back-bonding. When **7′**^–^ is oxidized to produce **7′**^•^ the bond length changes described above are reversed, an indication that the back-bonding in **7′**^–^ is reduced in **7′**^**•**^. Relative to **8′**, **7′**^•^ appears to have somewhat less backbonding.

**Figure 10:**
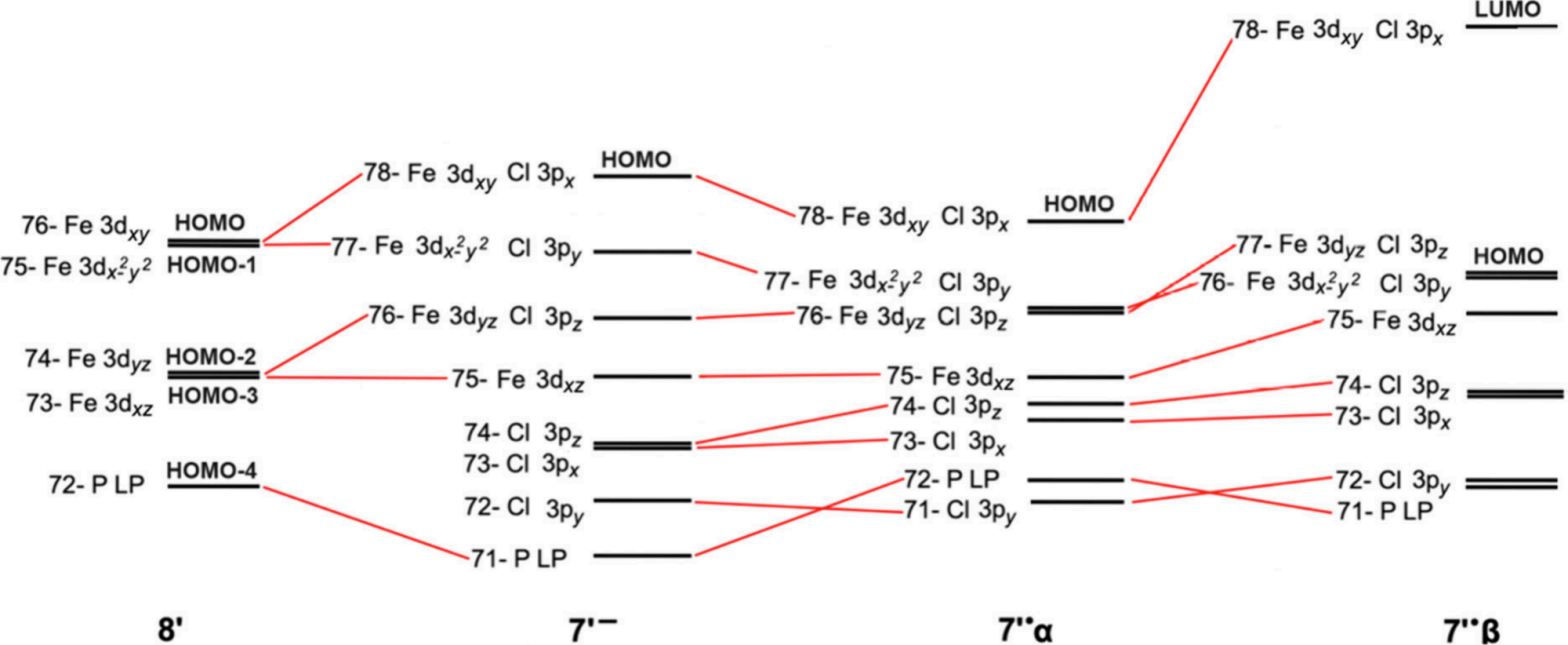
MO diagrams for model *trans* PMe_3_ complexes **8′**, **7′**^–^, and **7′**^•^. The orbitals are depicted in Table 5. The orbitals energies of **7′**^•^α, **7′**^–^, **8′** are aligned at HOMO–3 level, as this is the Fe 3d_*xz*_ MO that does not interact directly with any Cl orbitals. In spite of the reduction in symmetry, this figure maintains the *z* axis as the P–Fe–P axis and the *x* axis as perpendicular to the FeP_2_Cl plane.

Table 5 compiles the upper valence molecular orbitals (MOs) for the three model complexes, and Figure 10 illustrates how the energies of the MOs evolve from the 18-electron tricarbonyl **8′** to **7′**^–^ and then **7′**^•^. It is helpful to include **7′**^–^ in this sequence as **7′**^•^ is an unrestricted calculation with different MOs for α and β spins, and **7′**^–^ shows how the chloride ligand splits the iron 3d orbitals before they are split further by the unrestricted calculation in **7′**^•^.

**Table 5:**
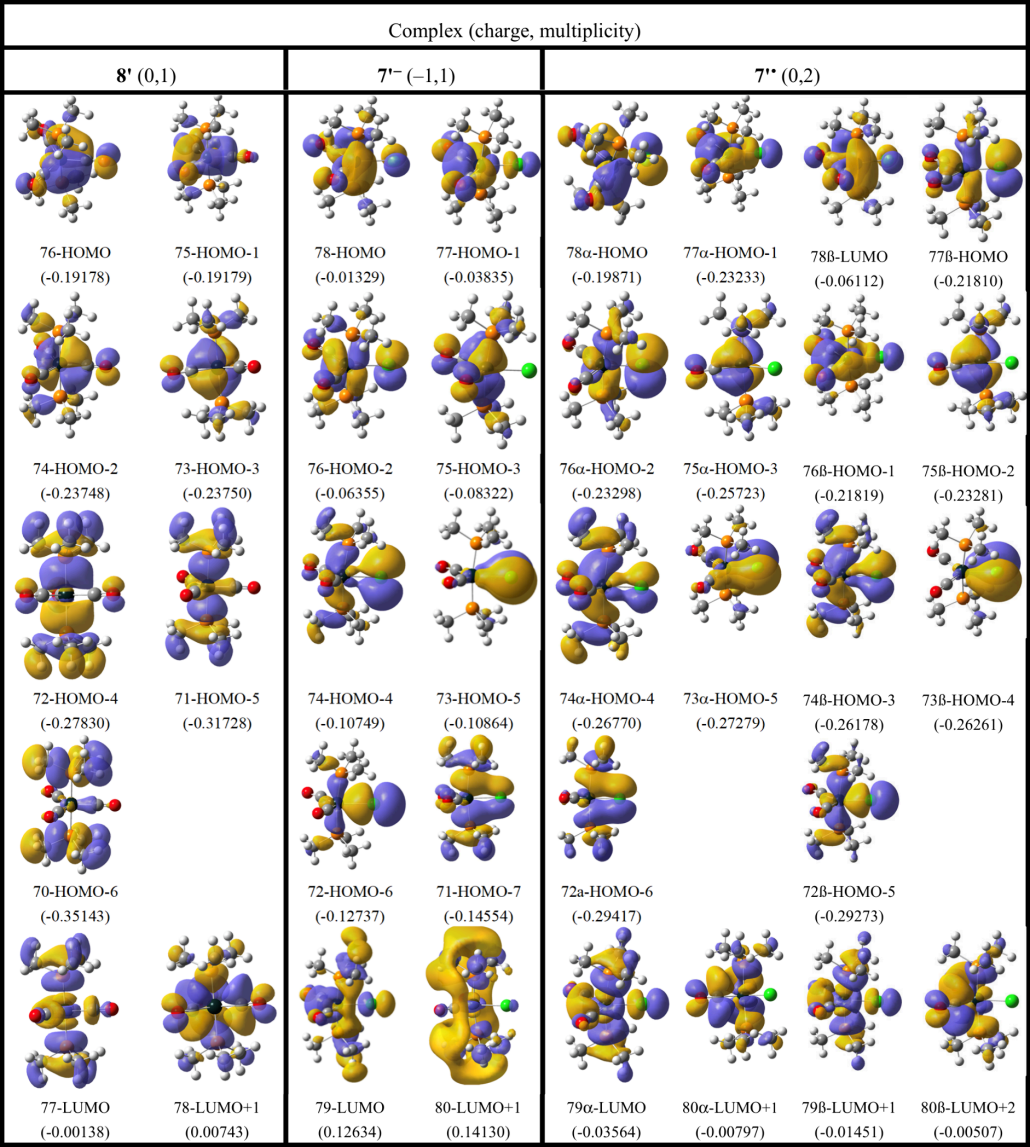
MOs of Model *trans* PMe_3_ Complexes **8′**, **7′**^–^, and **7′**^•^ with Energies in Atomic Units

As depicted in Figure 10, **8′** displays the typical symmetry and orbital distribution for a 3d^8^ trigonal-bipyramidal complex.^27^ The two most stable iron 3d MOs, HOMO–2 and HOMO–3, are only π bonding with the carbonyl ligands. The two higher energy iron MOs, HOMO and HOMO–1, are π bonding with some of the carbonyls and σ antibonding with others. When one carbonyl ligand is replaced with a chloride ligand, the degeneracy of both of these pairs is broken. Comparison of HOMOs of **8′** and **7′**^–^ demonstrates that the strong destabilization of two MOs in **7′**^–^ is due to the π antibonding character with the chloride ligand. When a β electron is removed from the HOMO of **7′**^–^, the MO splits and the energy of the β MO increases, becoming the LUMO of **7′**^•^β. All of the MOs in **7′**^•^β are higher in energy due to the loss of an exchange integral, but they follow the same order as **7′**^•^α.

Attention was next turned to the cyclic voltammetry data in Figures 5 and s10. First, consider the oxidation of **2a** by **1a**^+^ as expressed in eq 1:
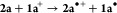
1

The sum of the *E*_1/2_ values for the half-reactions (+0.03 to +0.04 V) predict that it should be exergonic by −0.69 to −0.92 kcal mol^–1^, as calculated from the Nernst equation. Thus, the energies of each of the four species (i.e., **2a′**/**1a′**^+^/**2a′**^•+^/**1a′**^•^) were calculated as described in the Experimental Section. As summarized in Table 6, when the geometries were optimized with solvent corrections, the reaction was computed to be exergonic in CH_2_Cl_2_ by −0.68 kcal mol^–1^ in accord with the experimental data. However, the process was predicted to be slightly endergonic in the gas phase and to have mild solvent dependency. In spite of **1a′**^+^ having only 16 valence electrons, it has a singlet ground state because the strong π donation from the Cl destabilizes the HOMO of **1a′**^•^ such that removal of its remaining electron offsets the alternative of removing the electron from d_*x*^2^__–__*y*^2^_ orbital that is strongly stabilized by π-back-bonding of the two CO ligands.

**Table 6:** Computational Data^a^ for the Oxidation of **2a′** by **1a′**^+^ (See eq 1)^b^

description	kcal mol^–1^	*E*_cell_
TPSS gas	+0.53	–0.023
TPSS-SMD correction; CH_2_Cl_2_	+0.10	–0.004
TPSS-SMD correction; MeCN	–0.19	+0.008
TPSS-SMD optimization; CH_2_Cl_2_	–0.68	+0.029

a The ground state of **1a′**^+^ is a singlet; see Table s2.

b Experimental range: –0.69 to −0.92 kcal mol^–1^.

**Figure 11:**
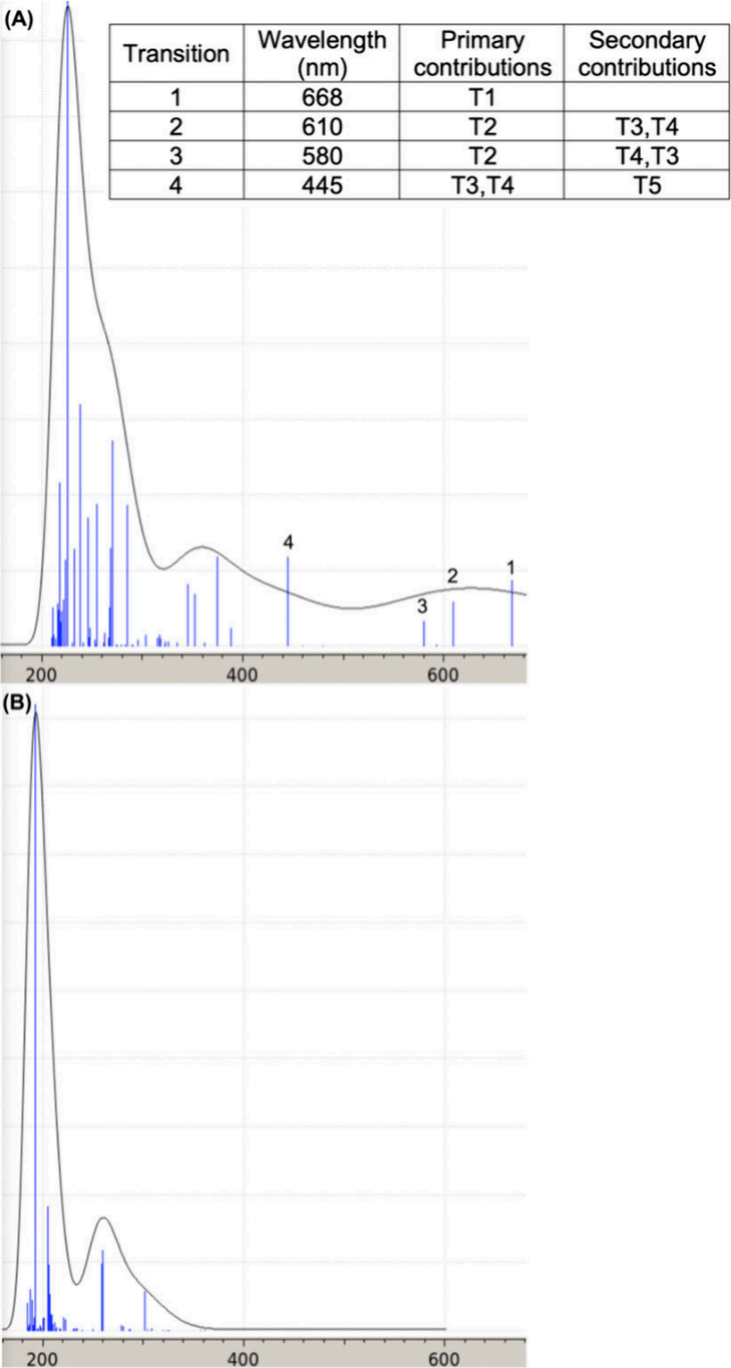
TD-DFT UV–visible spectra for (A) **1a′**^•^ and (B) **2a′**. The visible transitions are mainly due to the minority spin: 1 is mainly HOMO–1 to LUMO, 2 and 3 are mainly HOMO–2 to LUMO, and 4 is HOMO to LUMO+1; see Table s3 for details.

Next, attention was focused on modeling the UV–visible spectra in Figure 3 with TD-DFT. The spectra computed for **1a′**^•^ and **2a′** are depicted in Figure 11. No intensity is observed for **2a′** in the visible region, consistent with the white to pale yellow color of **2a** and its experimental UV–visible spectrum (Figure 3). However, **1a′**^•^ exhibits four moderately strong absorptions in the blue and orange/red regions, consistent with the green color of **1a**^•^ and its UV–visible spectrum. As expected, the PMe_3_-substituted model complexes **7′**^**•**^ and **8′** show very similar spectra, as illustrated in Figure s11.

## Discussion

### Mechanism of Formation of **1a**^•^

**Scheme 3:**
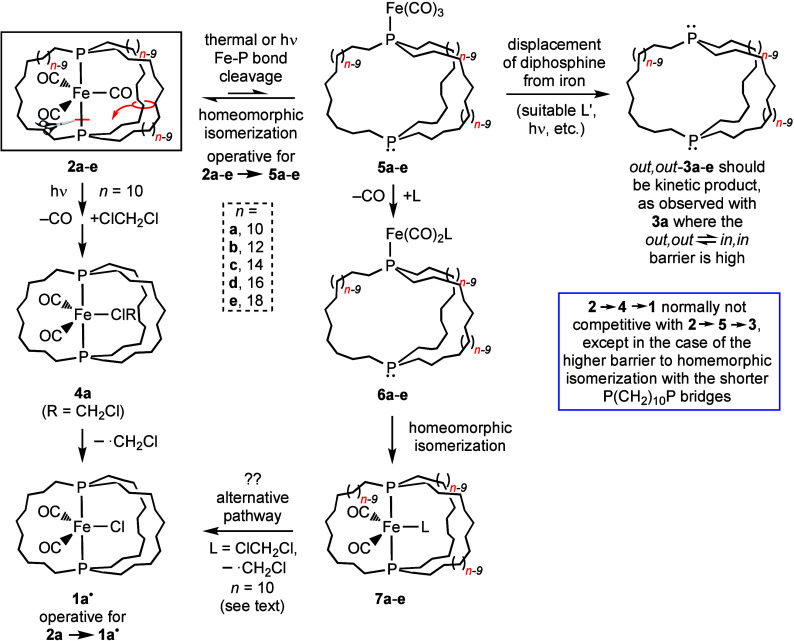
Mechanism Proposed for Conversions of the Iron Tricarbonyl Complexes **2a**-**e** to the Free Diphosphines **3a**-**e** and the Title Complex **1a**^**•**^

Most of the pentacoordinate 17-valence-electron iron(I) carbonyl radicals in Figure 1 were accessed by simple oxidative or reductive pathways. In contrast, the title complex **1a**^**•**^ is prepared by the photolysis of an iron carbonyl phosphine complex (**2a**) in the absence of conventional oxidizing or reducing agents (Scheme 1). Such photolyses can labilize either the carbonyl or phosphine ligands,^28^ and the dichloromethane solvent is the only possible source of the chlorine atom in **1a**^•^. Over the last 40 years, the coordination chemistry of dichloromethane has been extensively developed, evolving from conjecture^29^ to spectroscopically characterized intermediates^30^ to isolable adducts.^31^ Thus, the postulation of an initial adduct **4a** (Schemes 1 and 3) seems plausible.

This would be followed by inner sphere chlorine atom transfer by one of several mechanistic variants,^32^ generating the iron-centered radical **1a**^**•**^ and the carbon-centered radical ^•^CH_2_Cl. Indeed, many dichloromethane complexes undergo carbon–chlorine bond cleavage, but with the next observable being an addition product of the type L_*y*_M(Cl)(CH_2_Cl).^30b^ However, this might be suppressed by the cage-like diphosphine ligand. An experimental and computational study involving the transient 18-valence-electron complex (η^5^-C_5_H_5_)Re(CO)_2_(ClCH_2_Cl) implicated subsequent thermal conversion to the 17-valence-electron species [(η^5^-C_5_H_5_)Re(CO)_2_(Cl)]^•^.^33^

None of the other dibridgehead diphosphine complexes **2b**-**e** (Scheme 1) give an analogous iron(I) chloride complex upon photolysis. As shown in Scheme 3, the mechanism of demetalation of **2a**-**e** to the free dibridgehead diphosphines **3a**-**e** is believed to involve initial iron–phosphorus bond cleavage and homeomorphic isomerization to yield the η^1^-diphosphine adducts **5a**-**e**. Since **2a** has the smallest diphosphine cage, its conversion to **5a** would be expected to be slower. Indeed, the barriers to isomerization from *out,out*-**3a**,**b**,**c** to *in,in*-**3a**,**b**,**c** decrease from 27.6 to 14.9 to 13.7 kcal mol^–1^ (Δ*G*^⧧^_353 K_).^12^ Thus, it would be no surprise if CO/CH_2_Cl_2_ photosubstitution of **2a** to give the dichloromethane complex **4a** and then **1a**^**•**^ were to become competitive (Scheme 3).

A less direct pathway to **1a**^**•**^ would involve a CO/CH_2_Cl_2_ substitution of **5a**, followed by intermediates of the types **6a** and **7a** (Scheme 3). If this sequence were operative, it would seem likely that the **5b**-**e** generated from **2b**-**e** would undergo analogous substitutions, and ultimately form some **1b**-**e**^•^ as byproducts. However, there has been no evidence for the generation of significant quantities of these species.

### Redox Properties of **1a**^•^

As illustrated by the cyclic voltammograms in Figures 6 and s10, **1a**^•^ can undergo a partially reversible oxidation to the 16-valence-electron cation **1a**^+^ in CH_2_Cl_2_. There is seemingly the possibility for dichloromethane coordination to **1a**^+^ as well. The octahedral coordination geometry that would result has abundant precedent.^11a,34,35^ Interestingly, no evidence was seen for reduction of **1a**^•^, including cathodic scans out to −2.0 V, close to the solvent-imposed limit. Reduction would presumably yield **1a**^–^, an 18-valence-electron species analogous to computationally characterized **7′**^–^.

The *E*_1/2_ value for the couple **1a**^•^/**1a**^+^ (0.020 V) is also a puzzle. This is only slightly greater than that for the tricarbonyl complexes **2a**/**2a**^+^ (−0.010 V), each of which have one additional valence electron. Nonetheless, the oxidation of **1a**^**•**^ is only slightly less thermodynamically favorable than **2a**, consistent with the DFT data in Table 6. As shown in Figure 10, when the strong π acceptor, CO, is replaced by the strong π donor, Cl^–^,^36^ two of the iron d orbitals are markedly destabilized. The HOMO is so strongly destabilized that the 17-electron neutral species becomes more stable than the 18-electron anion. Loss of an electron from the HOMO of the 18-electron anion stabilizes the remaining electron in this MO; now its orbital energy is nearly identical to that of the HOMO of the 18-electron tricarbonyl system. Accordingly, the *E*_1/2_ value for the **1a**^•^/**1a**^+^ couple is only slightly different from that of the **2a**/**2a**^+^ couple. However, the *E*_1/2_ values for couples involving higher homologues of the tricarbonyl complexes, **2b**/**2b**^+^ and **2c**/**2c**^+^, indicate much more thermodynamically favorable oxidations (−0.136 and −0.146 V).^11a,37^ Thus, there is a significant cage size effect.

### Other Pentacoordinate Iron(I) Radicals

Of the isolable iron carbonyl radicals in Figure 1, the four reported by Berke,^6^*trans*-[Fe(CO)_2_(Br)(P(O*i*Pr)_3_)_2_]^•^, *trans*-[Fe(CO)_2_(I)(P(O*i*Pr)_3_)_2_]^•^, *trans*-[Fe(CO)_2_(Br)(PEt_3_)_2_]^•^, and *trans*-[Fe(CO)_2_(I)(PEt_3_)_2_]^•^, are the most relevant. All are especially air-sensitive as solids, whereas **1a**^•^ survives more than 6 h (Figure s12). The PEt_3_ adducts give IR ν_CO_ bands (X = Br, 1955/1884 cm^–1^; I, 1956/1886 cm^–1^) comparable to those of **1a**^•^ (1944/1861 cm^–1^). Using similar synthetic approaches as Berke, others later attempted the isolation of our computational model complex *trans*-[Fe(CO)_2_(Cl)(PMe_3_)_2_]^•^ (**7′**^•^).^7^ The green material, characterized by IR (1984/1935 cm^–1^), was reported to be thermally sensitive and rapidly react with air, and could never be obtained in pure form. These observations vividly illustrate the stabilization imparted by the dibridgehead diphosphine cage of **1a**^•^. Coincidentally, Berke has also studied **7′**^•^ by DFT, but communicated only a few structural parameters.^38^

Berke also reported the EPR spectra of his four radicals, as well as P(OMe)_3_ analogs that could not be isolated in pure form. The two PEt_3_ adducts give g and A_0_ values (2.057/2.55 and 2.078/2.40) very close to those of **1a**^•^ (2.05/2.48). The radical *trans*-[Fe(CO)_2_(I)(PEt_3_)_2_]^•^ could furthermore be crystallographically characterized, and extended Hückel calculations were reported for the PH_3_ analog.^6^ In several cases, Berke could reduce his radicals with Na/Hg under nitrogen and obtain diamagnetic adducts with Fe–N_2_–Fe linkages. Under similar conditions using sodium, no reaction was observed with **1a**^•^, in parallel with the cyclic voltammetry data.

The Mössbauer data for **1a**^**•**^ and **2a** in Figure 5 can be compared to those of the 18-valence-electron complexes *trans*-Fe(CO)_3_(L)_2_ (L = CO, PPh_3_, PCy_3_), as summarized in Tables 3 and 4. However, among the iron-based radicals in Figure 1, only [Fe(CO)_5_]^•+^Al(OC(CF_3_)_3_)_4_^–^ has been characterized by Mössbauer spectroscopy. In all cases, the DFT calculations give quadrupole splittings (QS, Table 3) and isomer shifts (IS, Table 4) that are in excellent agreement. Also, the computational data for the PMe_3_ model compounds are very close to those of their experimental counterparts.

The most interesting Mössbauer comparisons involve the data for **1a**^•^ and **2a** versus those of [Fe(CO)_5_]^•+^Al(OC(CF_3_)_3_)_4_^–^ and Fe(CO)_5_. In both pairs of complexes, the paramagnetic species exhibits a dramatically lower quadrupole splitting (0.29 vs 2.38 mm s^–1^ and 0.53 vs 2.55 mm s^–1^), with that of **1a**^**•**^ being conspicuously small. The larger splitting of the diamagnetic iron(0) complexes arises from the d^8^ configurations, which, as compared to a spherical d^10^ atom, have less electron density along the P–Fe–P axis. When another electron is lost from the d orbital in the (CO)_2_FeCl plane to give iron(I), the electron density becomes more spherical, reducing the quadrupole splitting.

Also, the isomer shifts of the paramagnetic iron(I) complexes are greater than those of their diamagnetic iron(0) counterparts (0.14 vs −0.12 mm s^–1^ and 0.17 vs −0.08 mm s^–1^, respectively). This is opposite to some literature generalizations regarding oxidation state trends [e.g., Fe(0) > Fe(I)].^22b,23^ However, fuller treatments highlight the independent roles of ligand fields and covalency, coordination numbers, and bond lengths.^22b,23a^ Furthermore, Peters has reported an iron carbonyl triphosphine system that can be isolated in three oxidation states, and his isomer shift trend parallels ours [Fe(II) > Fe(I) > Fe(0)].^9c^ The doublet character and (in the case of **1a**^**•**^) the strong donation from the chloride ligand would be expected to enhance isomer shifts. So to sum, pairs of closely related pentacoordinate paramagnetic iron(I) and diamagnetic iron(0) complexes appear to give diagnostically different isomer shifts and quadrupole splittings.

### Overview and Conclusion

**Figure 12:**
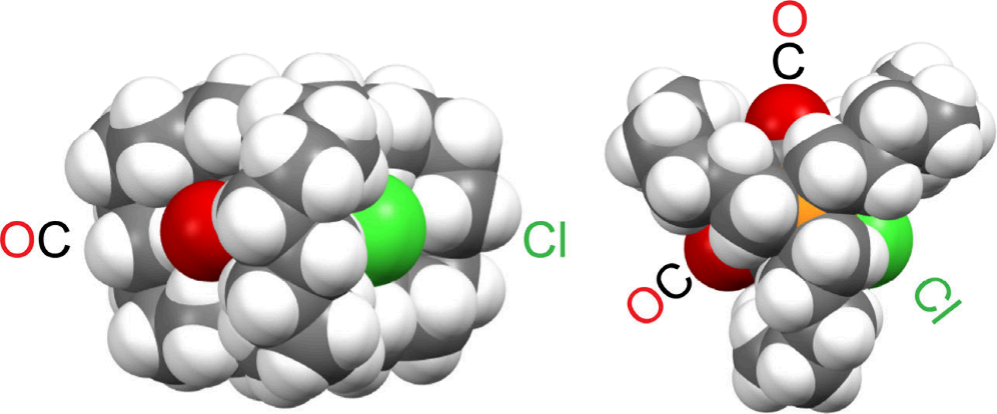
Space-filling representations of **1a**^•^.

The steric stabilization afforded by the dibridgehead diphosphine cage of the radical **1a**^•^ can also be visualized with space-filling representations, as shown in Figure 12. The equatorial CO and Cl ligands are visible only in a “peekaboo” mode, and the tightly fitting methylene chains strongly shield the metal. Despite these favorable factors, at least two potential Achille’s heels remain. One is direct air oxidation (Figure s12), presumably by some outer sphere process. Another would be homeomorphic isomerization, a pathway we consider operative for isosteric **2a** as outlined in Scheme 3. This would expose the iron atom, facilitating a variety of possible degradation reactions. For this reason, related complexes with less conformationally flexible phosphorus–phosphorus linkages have been a long-standing synthetic goal of one author.^39^

In conclusion, this work has established a new strategy for the stabilization of organometallic radicals based upon the steric shielding provided by cage-like *trans*-spanning dibridgehead diphosphine ligands. Importantly, these ligands can also accommodate square planar^35,40^ and octahedral^11a,34,35^ coordination geometries, so this approach could have considerable generality. However, such efforts will be facilitated by the development of other synthetic routes, as that used for **1a**^•^ (Schemes 1 and 3) is only feasible for one cage size. Nonetheless, the different reaction modes of **2a**-**e** as a function of cage size provide valuable insight regarding homeomorphic isomerization in coordination chemistry. It is also easy to envision substituted dibridgehead diphosphines that provide even more steric shielding for the L_*y*_M core, and the analogous dibridgehead diarsines and distibines are also available.^13^

## Safety Statement

***Caution!** The 450 W photochemical lamp emits considerable heat during use, requiring an external cooling well.^12,13^ Interruption of the water flow in the quartz cooling well can lead to glass failure, solvent ignition, and other potential hazards. Attached hosing should be checked thoroughly before operation of the lamp. Ultraviolet light produced by the lamp is damaging to biological tissues. When in use, proper PPE should be worn (e.g., UV protective goggles/glasses, lab coat, gloves) to ensure that exposure is minimized. Care should also be taken when using liquid nitrogen for Schlenk line traps to avoid condensation of liquid oxygen from air.*

## Experimental Section

### General Procedures

Reactions and workups were conducted under inert atmospheres. Chemicals were treated as follows: hexanes, CH_2_Cl_2_, and toluene, dried and degassed using a Glass Contour solvent purification system; PMe_3_ (Strem, 98%) used as received; silica gel (40–63 μm mesh, Silicycle) flame-dried and left under vacuum for 1 day before use.

### *trans*-Fe(CO)_2_(Cl)(P((CH_2_)_10_)_3_P) (**1a**^•^)

A flame-dried Schlenk flask was charged with *trans*-Fe(CO)_3_(P((CH_2_)_10_)_3_P) (**2a**; 0.278 g, 0.446 mmol), PMe_3_ (0.69 mL, 6.69 mmol), and CH_2_Cl_2_ (10 mL), and placed in front of a water-cooled quartz immersion well of a Hanovia 450 W lamp. As illustrated with photographs elsewhere,^12,13^ the sample was irradiated overnight with stirring. The solvent was removed by an oil pump vacuum. The residue was dissolved in hexanes and applied to a small pipet column of silica gel. The column was rinsed with hexanes (eluting **3a**) and then CH_2_Cl_2_. The dark green fractions were collected, and the solvent was removed by an oil pump vacuum. The residue was washed several times with hexanes and dried under vacuum to give **1a**^•^ (0.088 g, 0.140 mmol, 31%) as a green solid, dec pt. 166 °C (open capillary). Anal. Calcd for C_32_H_60_ClFeO_2_P_2_ (630.07): C, 61.00; H, 9.60. Found: C, 61.24; H, 9.76.

IR (powder film, cm^–1^): 2922 (m), 2852 (m), 1944 (s, ν_C≡O_), 1861 (s, ν_C≡O_), 1456 (w), 1259 (w), 1074 (w), 1017 (w), 948 (w), 804 (w), 713 (m). HRMS (ESI, *m*/*z*): calcd for C_30_H_60_P_2_FeCl [M – 2CO]^+^: 573.3203, found: 573.3198; calcd for C_32_H_61_P_2_FeO_2_ [M – Cl + H]^+^: 595.3491, found: 595.3480.

### Computations

Input models were built with Avogadro software. Except as otherwise noted the density functional theory (DFT) calculations were performed with the Gaussian 16 program (revision C.01).^41^ Geometry optimizations and frequency calculations in the gas phase used the B3LYP^42^ functional and the 6-311G(d)^43^ basis set for all atoms. Tight convergence criteria were used for the optimizations. Wave function stability calculations were performed to confirm the absence of lower-energy numerical solutions for all computed structures. Ultrafine grids (99,590 points per atom) were used as implemented in the Gaussian software. Calculations involving redox phenomena were performed with TPSS^44^ functional with the 6-311G(d) basis set for all atoms. The TPSS functional has performed well for predicting structures and electrochemical properties of other iron and nickel complexes.^45^ Initially, dichloromethane and acetonitrile solvent corrections with SMD^46^ were applied to the gas-phase-optimized geometries. The solvent dependency suggested that optimizing the structure in CH_2_Cl_2_ would bring its predicted value closer to the experimental one. Mössbauer calculations were done in the gas phase with B3LYP/def2-TZVP^47^ method/basis set combination in the Orca 5.0.3 software package,^48^ as recommended.^23a^

### Crystallography

#### A

A small quantity of **1a**^•^ was suspended in hexanes, and CH_2_Cl_2_ was added dropwise until the mixture was homogeneous. The sample was allowed to slowly concentrate under argon at −38 °C. After 5 d, green prisms were collected and data obtained per Table 1. Cell parameters were obtained from 90 data frames taken at widths of 1° and refined with 70838 reflections. Integrated intensity information for each reflection was obtained by reduction of the data frames with the program APEX3.^49^ Lorentz, polarization, and absorption corrections were applied, the last using the program SADABS.^50^ The space group was determined from systematic reflection conditions and statistical tests. The structure, a hexane hemisolvate, was solved using XT/XS in APEX3^49,51^ and refined (weighted least-squares refinement on *F*^*2*^) to convergence.^51,52^ Elongated ellipsoids and nearby residual electron density peaks on the methylene chains (C1–C10, C11–C20, and C21–C30) suggested disorder, which was successfully modeled. Further nearby residual electron density peaks, and the larger thermal ellipsoids of C21–C30, indicated additional disorder, but no efforts were made to model this. The occupancy ratios of disordered atoms were first refined individually. Since they were close, they were grouped together, giving a final 59:41 ratio. Appropriate restraints and constraints were added to keep the bond distances, angles, and thermal ellipsoids meaningful. All non-hydrogen atoms were refined anisotropically. Hydrogen atom positions were calculated and refined using a riding model.

#### B

A hexane/toluene (2:1 v/v) solution of **2a** was kept at −20 °C. After 3 d, a yellow block-shaped crystal was collected, which was cut as it appeared to be a multi twin or cracked. Data were obtained as per Table 1. Crystal screening, unit cell determination, and data collection were carried out using a XtaLAB Synergy, Dualflex, HyPix diffractometer. The diffraction pattern was indexed and the total number of runs and images was based on the strategy calculation from CrysAlisPro,^53^ which was used throughout. The unit cell was refined using 45684 reflections. Integrated intensity information for each reflection was obtained by reduction of data frames within the same software suite, and Gaussian and numerical absorption corrections were similarly applied. A hexane molecule was found, with the C–C midpoint coincident with an inversion center. The occupancy was refined to 0.90, corresponding to 0.45 molecules of solvated hexane per iron atom. A residual electron density peak near C2s suggested disorder of the hexane, which was modeled between two positions with an occupancy ratio of 81:19. Also, elongated or abnormal thermal ellipsoids and/or residual electron density peaks was noted near all the carbon atoms in three hydrocarbon chains except C28. This disorder was modeled between two positions with an occupancy ratio of 54:46. Appropriate restraints and constraints were added to keep the bond distances and thermal ellipsoids meaningful. Systematic reflection conditions and statistical tests afforded the space group (Table 1), as confirmed by ShelXT 2018/2^54^ using dual methods and Olex2–1.5.^52^ The structure was refined by full matrix least-squares minimization on *F*^2^ using version 2019/1 of XL.^51^ All non-hydrogen atoms were refined anisotropically. Hydrogen atom positions were calculated and refined using a riding model.

## Supporting Information Available

Additional instrumental procedures, spectroscopic, electrochemical, and computational data, and photographs of samples (PDF)
