# Supplementary material for: A Caged Neutral 17-Valence-Electron Iron(I) Radical [Fe(CO)2(Cl)(P((CH2)10)3P)]•: Synthetic, Structural, Spectroscopic, Redox, and Computational Studies
Source: Inorg Chem. 2024 Aug 20;63(35):16313–26. doi: 10.1021/acs.inorgchem.4c02275 (PMC11379347; doi:10.1021/acs.inorgchem.4c02275)
Supplement: Supplementary file 1 — ic4c02275_si_001.pdf [file ic4c02275_si_001.pdf]

A Caged Neutral Seventeen-Valence-Electron Iron(I) Radical $[Fe(CO)_2(Cl)(P((CH_2)_{10})_3P)]^{\bullet}$: Synthetic, Structural, Spectroscopic, Redox, and Computational Studies

Samuel R. Zarcone,[a] Zihan Zhang,[b] Suhashini Handunneththige,[a] Zhen Ni,[a] Nattamai Bhuvanesh,[a] Michael Nippe,*[a] Karsten Meyer,*[b] Michael B. Hall,*[a] and John A. Gladysz*[a]

[a]Department of Chemistry, Texas A&M University, PO Box 30012, College Station, Texas 77842-3012, USA

and

[b]Department of Chemistry und Pharmacy, Inorganic Chemistry, Friedrich-Alexander-Universität Erlangen-Nürnberg (FAU), Egerlandstraße 1, 91058 Erlangen, Germany

corresponding author email: gladysz@mail.chem.tamu.edu

General Methods. IR spectra were recorded on a Shimadzu IRAffinity-1 instrument with a Pike MIRacle ATR system (diamond/ZnSe crystal). UV-visible spectra were recorded on a Shimadzu UV-1800 spectrometer. Mass spectra were collected on a Thermo Scientific Q Exactive Focus instrument. Melting points were recorded using a Stanford Research Systems MPA100 (OptiMelt) automated apparatus. Microanalyses were conducted by Atlantic Microlab.

EPR Spectroscopy (Figures 4, s2-s5). Spectra were recorded on a JEOL continuous wave spectrometer JES-FA200, equipped with an X-band Gunn diode oscillator bridge, a cylindric mode cavity, and a helium cryostat. Measurements were carried out in solution or solid state under nitrogen atmospheres in quartz glass EPR tubes at 95 K or 293 K. The spectra were recorded using the following parameters: microwave frequency = 8.959 GHz, modulation amplitude 0.01 mT, microwave power 1.0 mW, modulation frequency 100 kHz, time constant of 0.1 s. Data analysis and simulation were carried out using the software "eview" and "esim", written by Dr. Eckhard Bill (MPI CEC, Mülheim/Ruhr),[s1,s2] on the basis of a spin-Hamiltonian description of the electronic ground state:

$$\hat{H} = D\left(\hat{S}_z^2 - \frac{1}{3}S(S+1) + \frac{E}{D}\left(\hat{S}_x^2 - \hat{S}_y^2\right)\right) + \mu_B \underline{g}\vec{S}$$

Here, S represents the total spin quantum number of the coupled system, D and E/D are the axial and rhombic zero-field parameters, respectively, and $\underline{g}$ is the g-matrix. Calculations are based on the $S = 5/2$ routines developed by Gaffney and Silverstone.[s3] EPR line widths, W, are given in units of 10^{-4} cm^{-1}/GHz at full-width-half-maximum (FWHM).

Magnetism data (Figure 4). Microcrystalline and powdered samples (10.0-25.0 mg) were loaded into a polycarbonate gel capsule inside a plastic straw and data were collected on a Quantum Design MPMS-3 SQUID magnetometer. The DC moment was recorded in the temperature range of 2–300 K with an applied DC field of 1 T. The DC moment was converted into molar magnetic susceptibility (χ_M) using the following formula (with H = magnetic field, n = moles of substance):

$$\chi_M = \frac{DC\ moment}{H \cdot n}$$

The magnetic susceptibility values were corrected for core diamagnetism (χ_{dia}) of the sample using tabulated Pascal's constants.[s4] Effective magnetic moments (μ_{eff}) were calculated using the following formula (with temperature T):

$$\mu_{eff} = 2.828 \cdot \sqrt{(\chi_M - \chi_{dia}) \cdot T}$$

For data simulation and analysis, the program "JulX2", written by Dr. Eckhard Bill (MPI CEC, Mülheim/Ruhr) was used.[s5]

Zero-field ^{57}Fe-Mössbauer (Figure 5). Spectra were recorded on a WissEl Mössbauer spectrometer (MRG-500) at 77 K in constant acceleration mode, with ^{57}Co/Rh as the γ-radiation source. WinNormos for Igor Pro software was used for the quantitative evaluation of the spectral parameters (least-squares fitting to Lorentzian peaks). The minimum experimental line width was determined at 0.21 mm s^{-1} (full width at half maximum, FWHM). The sample temperature was controlled by an MBBC-HE0106 Mössbauer He/N_2 cryostat with an accuracy of +/– 0.3 K. Least squares fitting of the Lorentzian signals was carried out with the "Mfit" software, developed by Dr. Eckhard Bill (MPI CEC, Mülheim/Ruhr).[s6,s7] The isomer shifts were reported relative to α-iron reference at 300 K.

Cyclic Voltammetry. Figures 6, s6-s9: Traces were recorded using a GAMRY Ref600 potentiostat and recorded in an argon glovebox as described in Figure 6. Potentials were referenced to the ferrocene/ferrocenium couple measured using internally added cobaltocene. Figure s10: Traces were recorded using a BASiEpsilon Electrochemical Workstation (Cell Stand C3) with the program Epsilon EC (version 2.13.77). Samples were prepared under N_2, and data were recorded as described in Figure s10 (ferrocene was added after each measurement).[s8]

Table s1. Atomic charges calculated with QTAIM.

**8'**		**7'•**	
**1 Fe**	0.97	**1 Fe**	1.07
**2 P**	1.69	**2 Cl**	–0.75
**3 O**	–1.17	**3 P**	1.67
**4 C**	0.68	**4 P**	1.67
**5 C**	–0.55	**5 O**	–1.15
**6 C**	–0.55	**6 O**	–1.15
**7 C**	–0.55	**7 C**	0.73
**8 P**	1.69	**8 C**	0.73
**9 C**	–0.55	**9 C**	–0.56
**10 C**	–0.55	**10 C**	–0.56
**11 C**	–0.55	**11 C**	–0.54
**12 O**	–1.18	**12 C**	–0.54
**13 C**	0.69	**13 C**	–0.56
**14 O**	–1.17	**14 C**	–0.56
**15 C**	0.68	**15 H**	0.02
**16 H**	0.01	**16 H**	0.01
**17 H**	0.03	**17 H**	0.07
**18 H**	0.03	**18 H**	0.03
**19 H**	0.01	**19 H**	0.03
**20 H**	0.03	**20 H**	0.01
**21 H**	0.03	**21 H**	0.07
**22 H**	0.03	**22 H**	0.01
**23 H**	0.03	**23 H**	0.02
**24 H**	0.01	**24 H**	0.01
**25 H**	0.03	**25 H**	0.03
**26 H**	0.01	**26 H**	0.03
**27 H**	0.03	**27 H**	0.01
**28 H**	0.03	**28 H**	0.07
**29 H**	0.03	**29 H**	0.02
**30 H**	0.01	**30 H**	0.01
**31 H**	0.01	**31 H**	0.02
**32 H**	0.03	**32 H**	0.07
**33 H**	0.03		

Table s2. Computed relative energies of **1a'**$^{\cdot+}$ as a function of multiplicity.

Description	Multiplicity of 1a'$^{\cdot+}$	kcal/mol
B3LYP-gas-Gibbs free energy	1	0
	3	0.33
	5	10.45
TPSS-gas-single point-SCF energy	1	0
	3	8.87
	5	60.19
TPSSh-gas-single point-SCF energy	1	0
	3	5.20
	5	50.22
ωB97XD-gas-single point-SCF energy	1	0
	3	3.56
	5	38.27
BMK-gas-single point-SCF energy	1	0
	3	0.90
	5	33.21
MN15-gas-single point-SCF energy	1	0
	3	3.24
	5	40.34

Table s3. TD-DFT derived transitions and orbital representations for **1a'**$^{\cdot}$ and **7'**$^{\cdot}$.

Complex	Orbital contributions		Orbitals
**1a'**$^{\cdot}$	T1	169ß → 171ß (HOMO-1 → LUMO)	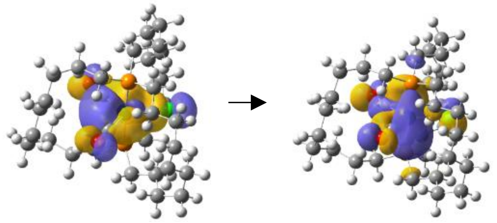
	T2	168ß → 171ß (HOMO-2 → LUMO)	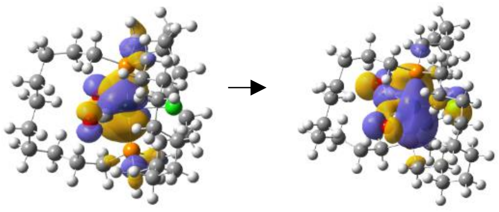
	T3	170ß → 172ß (HOMO → LUMO+1)	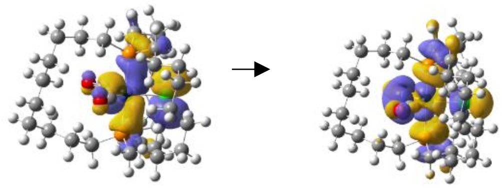

	T4	170α → 172α<br>(HOMO-1 → LUMO)	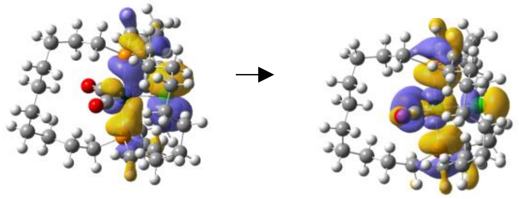
	T5	167α → 172α<br>(HOMO-4 → LUMO)	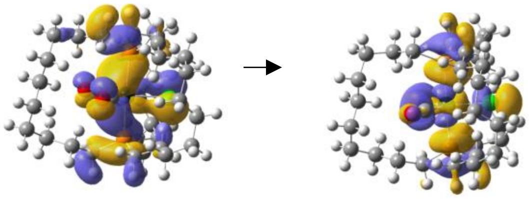
7'•	t1	76ß → 78ß<br>(HOMO-1 → LUMO)	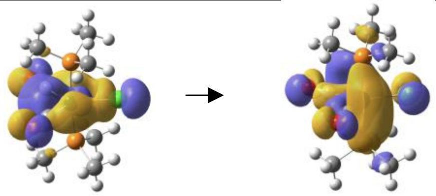
	t2	75ß → 78ß<br>(HOMO-2 → LUMO)	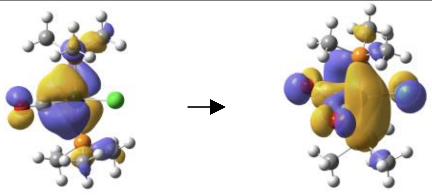
	t3	77ß → 79ß<br>(HOMO → LUMO+1)	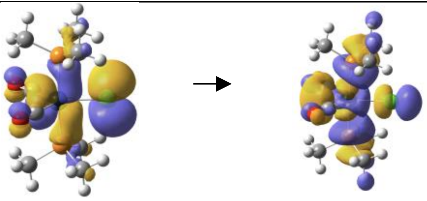
	t4	76α → 79α<br>(HOMO-2 → LUMO)	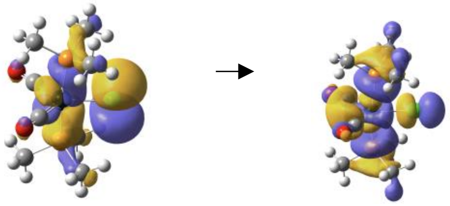
	t5	74α → 79α<br>(HOMO-4 → LUMO)	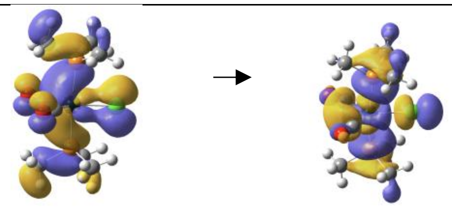

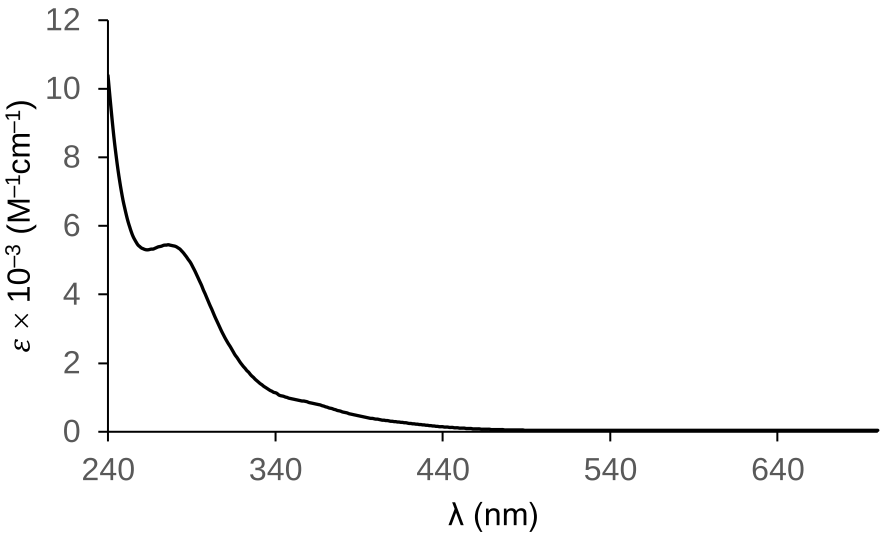

Figure s1. UV-visible spectrum of **2c** (9.50×10^{-5} M in CH_2Cl_2). λ_{max} (nm) [ε ($M^{-1}cm^{-1}$)]: 276 [5,360].

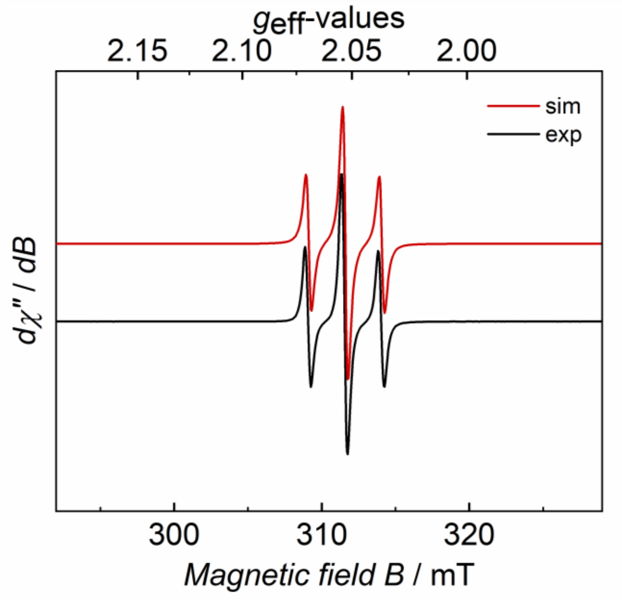

Figure s2. CW X-band EPR spectrum of **1a**$^{\bullet}$, recorded as a 3 mM dichloromethane solution at 293 K (black trace) and its simulation (red trace). Simulation parameters: effective spin $S = 1/2$, effective g-value $g_{iso} = 2.05$, linewidth $W_{FWHM,iso} = 0.39 \times 10^{-4}$ cm^{-1} / GHz. Voigt ratio (Lorentz = 0, Gauss = 1) $V_{iso} = 0$. Hyperfine coupling to two ^{31}P ($I = 1/2$, 100% nat. abundance) nuclei was determined as $A_{iso} = 23.7 \times 10^{-4}$ cm^{-1}.

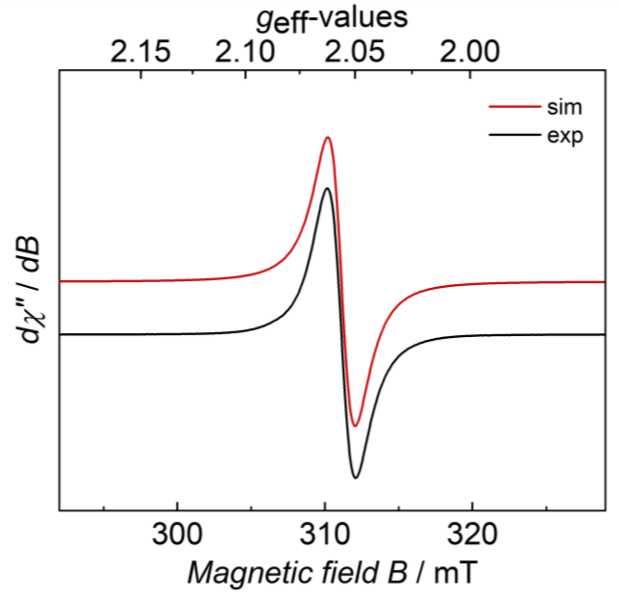

Figure s3. CW X-band EPR spectrum of **1a**$^{\bullet}$, recorded as a 3 mM frozen dichloromethane solution at 95 K (black trace) and its simulation (red trace). Simulation parameters: effective spin $S = 1/2$, effective g-value $g_{iso} = 2.05$, linewidth $W_{FWHM,iso} = 1.99 \times 10^{-4}$ cm^{-1} / GHz. Voigt ratio (Lorentz = 0, Gauss = 1) $V_{iso} = 0$.

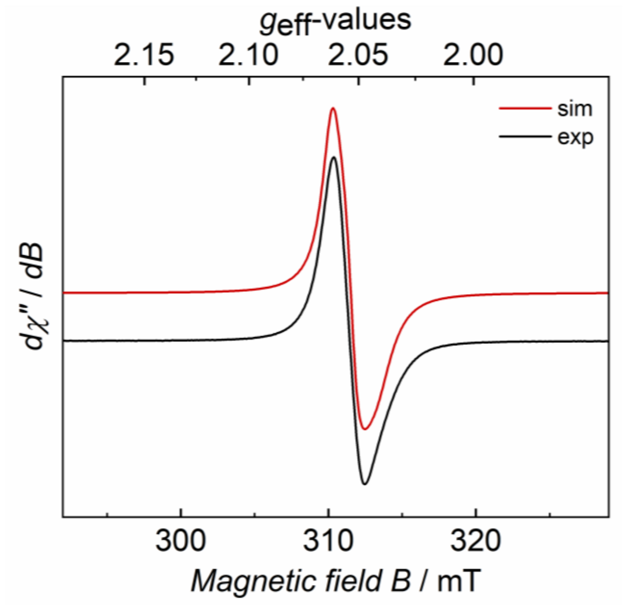

Figure s4. CW X-band EPR spectrum of **1a**$^{\bullet}$, recorded as a powdered sample at 95 K (black trace) and its simulation (red trace). Simulation shows a slightly rhombic $S = 1/2$ signal. Simulation parameters: effective g-values $g_1 = 2.06$, $g_2 = 2.05$, $g_3 = 2.04$, linewidths $W_1 = 0.95$ mT, $W_2 = 0.94$ mT, $W_3 = 1.72$ mT. Voigt ratio (Lorentz = 0, Gauss = 1) $V_1 = V_2 = V_3 = 0$.

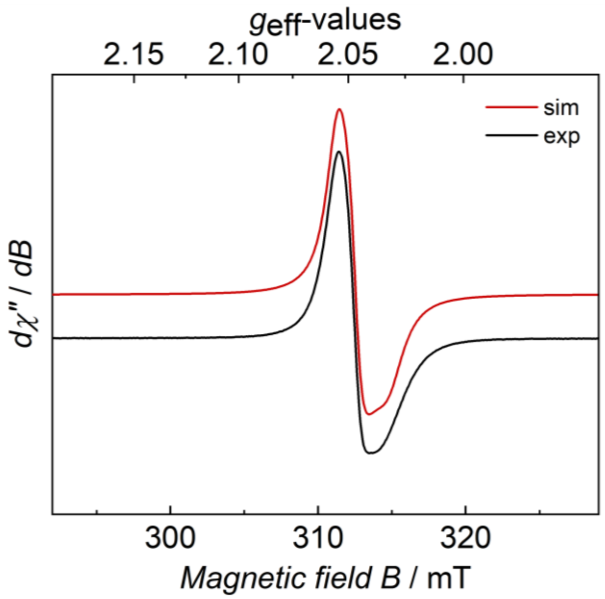

Figure s5. CW X-band EPR spectrum of **1a$^•$**, recorded as a crystalline sample at 95 K (black trace) and its simulation (red trace). Simulation shows a slightly rhombic $S = 1/2$ signal. Simulation parameters: effective g-values $g_1 = 2.06$, $g_2 = 2.05$, $g_3 = 2.03$, linewidths $W_1 = 1.12$ mT, $W_2 = 0.91$ mT, $W_3 = 1.62$ mT. Voigt ratio (Lorentz = 0, Gauss = 1) $V_1 = V_2 = V_3 = 0$.

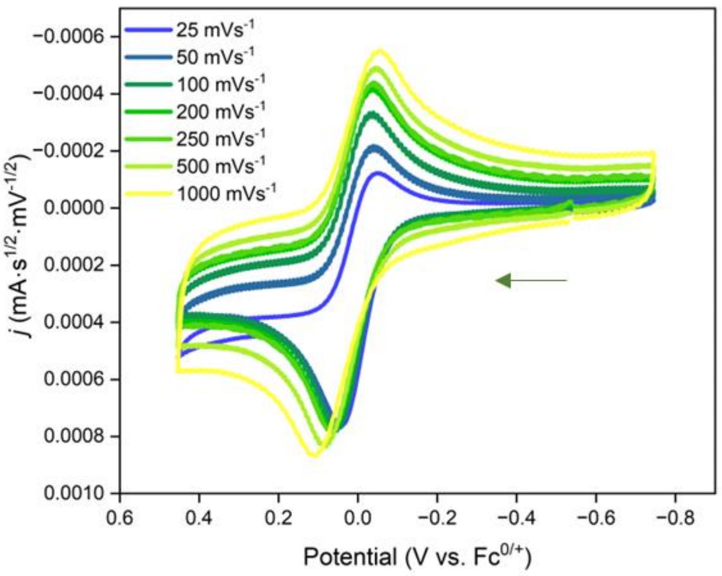

Figure s6. Cyclic voltammograms of **1a$^•$** at various scan rates under the conditions of Figure 6.

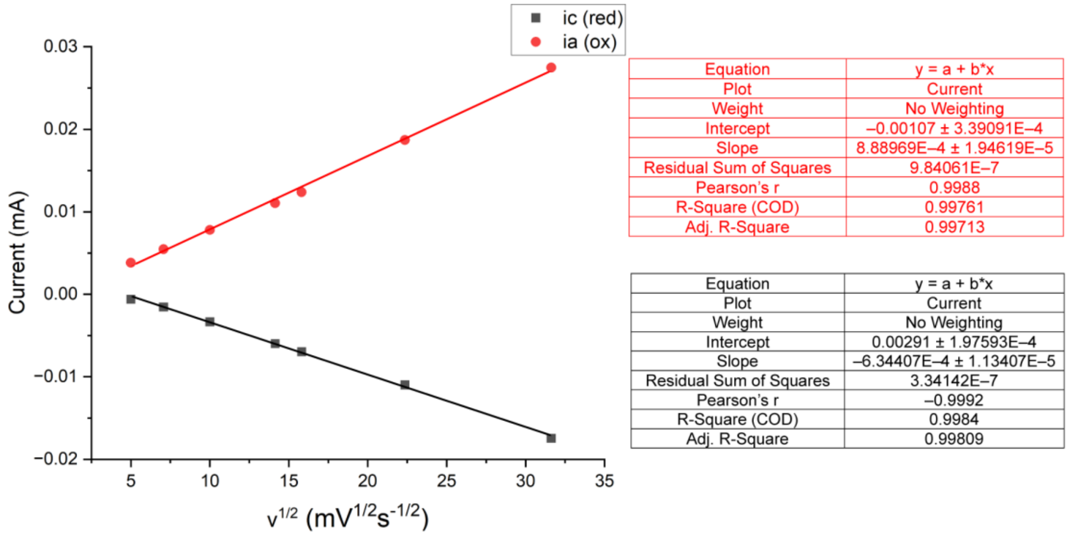

Figure s7. Scan rate dependence of cathodic and anodic peak current of the initial oxidation wave of the cyclic voltammograms of **1a**$^{\bullet}$ in Figure s6.

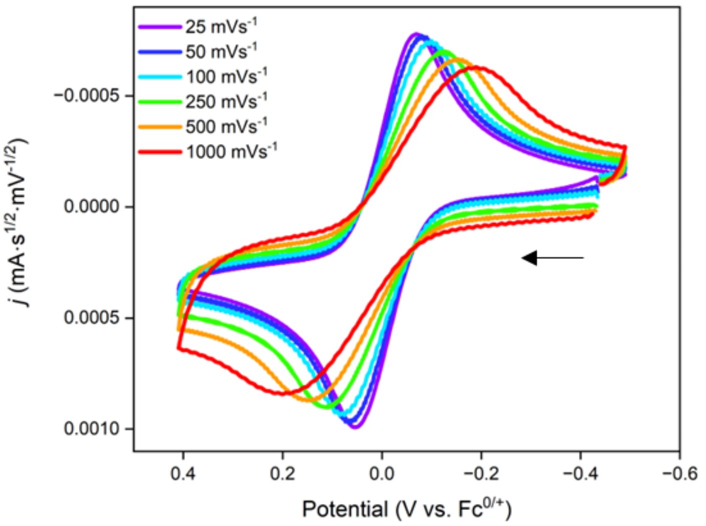

Figure s8. Cyclic voltammograms of **2a** at various scan rates at various scan rates under the conditions of Figure 6.

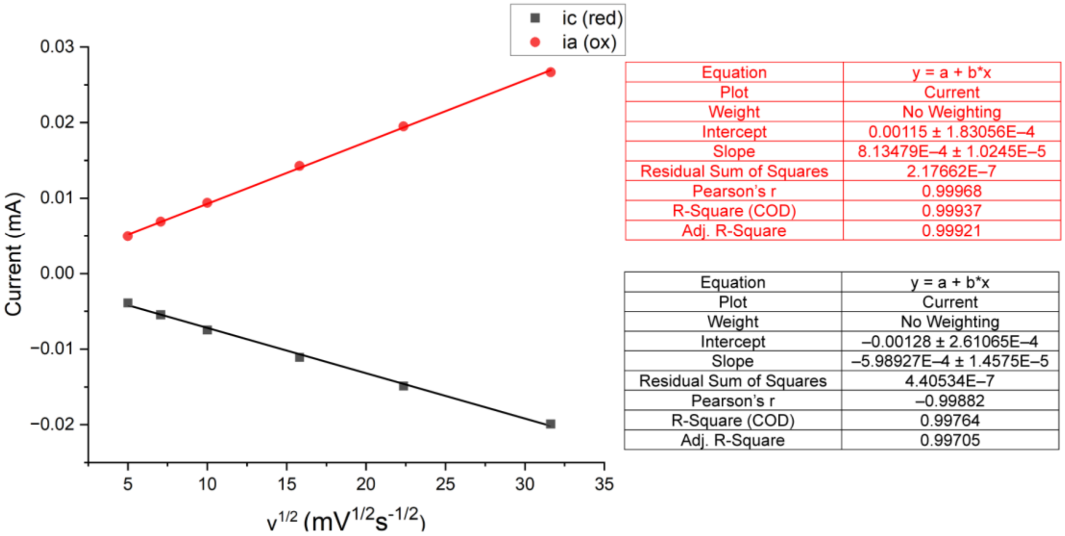

Figure s9. Scan rate dependence of cathodic and anodic peak current of the initial oxidation wave of the cyclic voltammograms of **2a** in Figure s8.

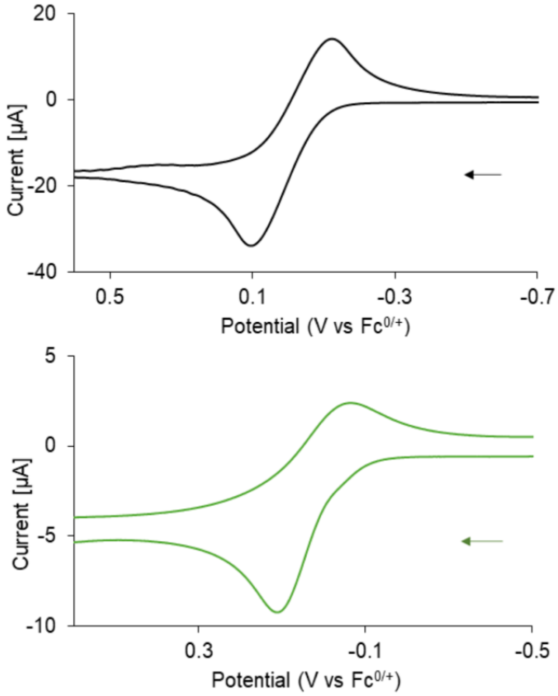

complex	$E_{p,a}$ (V)	$E_{p,c}$ (V)	$E_{1/2}$ (V)	ΔE (mV)	i_c/i_a
**1a•**	0.11	–0.07	0.02	179	0.70
**2a**	0.10	–0.12	–0.01	222	0.88

Figure s10. Additional cyclic voltammograms of **1a•** (top) and **2a** (bottom). Conditions: 0.0010 M in 0.10 M *n*-Bu_4N^+ PF_6^-/CH_2Cl_2, 23 ± 1 °C; 3 mm glassy carbon working electrode, Pt auxiliary electrode, and Ag/AgCl pseudoreference; scan rate, 200 mV/s; internal ferrocene = 0.00 V. All scans were continued to −2.0 and 1.0 V, but no additional features were observed.

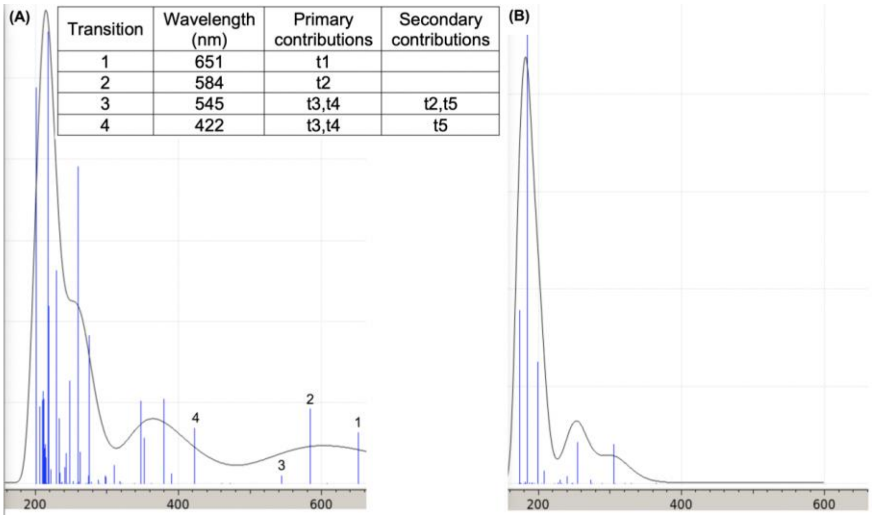

Figure s11. TD-DFT UV-visible spectra for model *trans* PMe_3 complexes (A) **7'**$^{\bullet}$ and (B) **8'**. Descriptions of transitions are shown in Table s3.

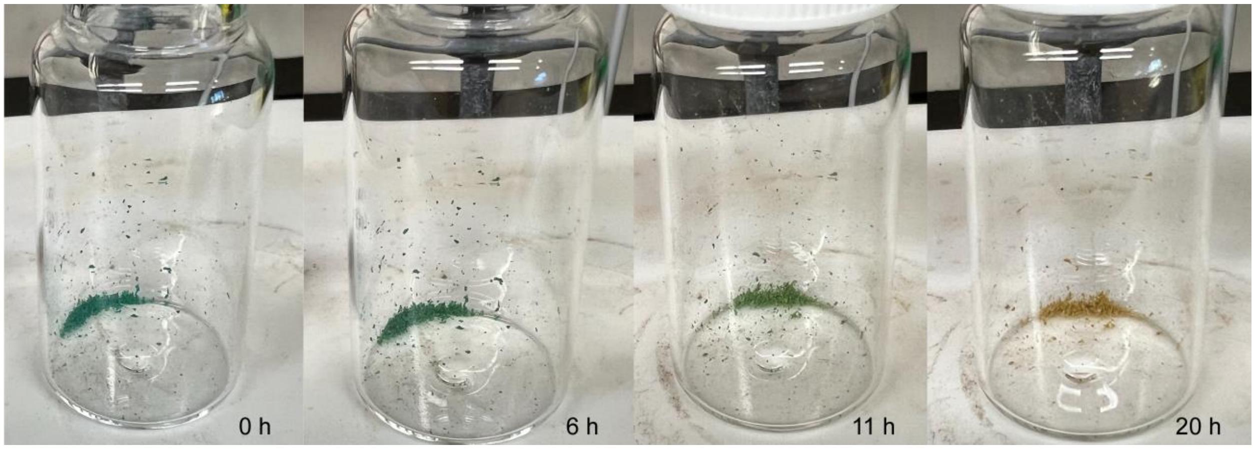

Figure s12. Gradual decomposition of **1a**$^{\bullet}$ under air at ambient temperature.

■ REFERENCES

(s1) Bill, E. *EPR Program eview*; MPI Kohlenforschung, Mülheim, Germany (2019).

(s2) Bill, E. *EPR Program esim*; MPI Kohlenforschung, Mülheim, Germany (2019).

(s3) Gaffney, B. J., Silverstone H. J. *Simulation of the EMR Spectra of High-Spin Iron in Proteins*, Springer, Boston, MA, 1993.

(s4) Bain, G. A.; Berry, J. F. Diamagnetic Corrections and Pascal's Constants. *J. Chem. Educ.* **2008**, *85*, 532–536.

(s5) Bill, E. *SQUID Program JulX2*; MPI Kohlenforschung, Mülheim, Germany (2019).

(s6) Bill, E. *Mössbauer Program Mcal*; MPI Kohlenforschung, Mülheim, Germany (2019).

(s7) Bill, E. *Mössbauer Program Mfit*; MPI Kohlenforschung, Mülheim, Germany (2019).

(s8) Connelly, N. G.; Geiger, W. E. Chemical Redox Agents for Organometallic Chemistry. *Chem. Rev.* **1996**, *96*, 877.
